# Fine Particulate Matter Induces Childhood Asthma Attacks via Extracellular Vesicle‐Packaged Let‐7i‐5p‐Mediated Modulation of the MAPK Signaling Pathway

**DOI:** 10.1002/advs.202102460

**Published:** 2021-11-23

**Authors:** Rui Zheng, Mulong Du, Man Tian, Zhaozhong Zhu, Chengcheng Wei, Haiyan Chu, Cong Gan, Jiayuan Liang, Renjie Xue, Fang Gao, Zhenguang Mao, Meilin Wang, Zhengdong Zhang

**Affiliations:** ^1^ Department of Genetic Toxicology The Key Laboratory of Modern Toxicology of Ministry of Education Center for Global Health School of Public Health Nanjing Medical University Nanjing 211166 China; ^2^ Department of Environmental Genomics Jiangsu Key Laboratory of Cancer Biomarkers, Prevention and Treatment Collaborative Innovation Center for Cancer Personalized Medicine Nanjing Medical University Nanjing 211166 China; ^3^ Department of Biostatistics Center for Global Health School of Public Health Nanjing Medical University Nanjing 211166 China; ^4^ Department of Respiratory Medicine Children's Hospital of Nanjing Medical University Nanjing 210008 China; ^5^ Department of Emergency Medicine Massachusetts General Hospital and Harvard Medical School Boston MA 02114 USA; ^6^ Key Laboratory of Environmental Medicine Engineering Ministry of Education of China School of Public Health Southeast University Nanjing 210009 China; ^7^ The Affiliated Suzhou Hospital of Nanjing Medical University Suzhou Municipal Hospital, Gusu School Nanjing Medical University Suzhou 215008 China

**Keywords:** PM_2.5_, childhood asthma, extracellular vesicles, let‐7i‐5p, MAPK signaling pathway

## Abstract

Fine particulate matter less than 2.5 µm in diameter (PM_2.5_) is a major risk factor for acute asthma attacks in children. However, the biological mechanism underlying this association remains unclear. In the present study, PM_2.5_‐treated HBE cells‐secreted extracellular vesicles (PM_2.5_‐EVs) caused cytotoxicity in “horizontal” HBE cells and increased the contractility of “longitudinal” sensitive human bronchial smooth muscle cells (HBSMCs). RNA sequencing showed that let‐7i‐5p is significantly overexpressed in PM_2.5_‐EVs and asthmatic plasma; additionally, its level is correlated with PM_2.5_ exposure in children with asthma. The combination of EV‐packaged let‐7i‐5p and the traditional clinical biomarker IgE exhibits the best diagnostic performance (area under the curve [AUC] = 0.855, 95% CI = 0.786–0.923). Mechanistically, let‐7i‐5p is packaged into PM_2.5_‐EVs by interacting with ELAVL1 and internalized by both “horizontal” recipient HBE cells and “longitudinal” recipient‐sensitive HBSMCs, with subsequent activation of the MAPK signaling pathway via suppression of its target *DUSP1*. Furthermore, an injection of EV‐packaged let‐7i‐5p into PM_2.5_‐treated juvenile mice aggravated asthma symptoms. This comprehensive study deciphered the remodeling of the extracellular environment mediated by the secretion of let‐7i‐5p‐enriched EVs during PM_2.5_‐induced asthma attacks and identified plasma EV‐packaged let‐7i‐5p as a novel predictor of childhood asthma.

## Graphical abstract

PM_2.5_ is considered an important type of allergen in childhood asthma. Let‐7i‐5p is packaged into EVs though an interaction with ELAVL1 and then internalized by recipient cells (HBE and sensitive HBSMCs). EV‐packaged let‐7i‐5p activate the MAPK signaling pathway by targeting *DUSP1*, resulting in cytotoxicity in “horizontal” recipient HBE cells and enhance contractility of “longitudinal” recipient‐sensitive HBSMCs.

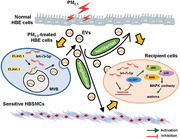

## 1 Introduction

Asthma is a common chronic respiratory disease characterized by airway hyperresponsiveness, airway inflammation and reversible airway remodeling.^[^ ^1^, ^2^ ^]^ Globally, asthma remains a serious health problem that affects 358 million individuals, especially among children.^[^ ^3^, ^4^ ^]^ Clinically, asthma attacks in children lead to reversible and repeated attacks of coughing, wheezing, shortness of breath, and chest tightness, thereby impairing quality of life and imposing a heavy burden on the family and community.^[^ ^5^ ^]^

As environmental pollution is predictable and preventable, airborne fine particulate matter <2.5 µm in diameter (PM_2.5_) has been considered a crucial type of allergen causing acute asthma attacks in children.^[^ ^5^, ^6^, ^7^ ^]^ Here, two airway cell types ultimately responsible for asthma pathogenesis are considered: human bronchial epithelial (HBE) cells, which form the first barrier between the external environment and the internal bronchus and are the major target of inhaled particles and pathogens,^[^ ^8^ ^]^ and human bronchial smooth muscle cells (HBSMCs) located below HBE cells, which are the main effectors of bronchial contraction and whose excessive contraction induces airway hyperresponsiveness.^[^ ^9^ ^]^ Emerging evidence has shown that various stimuli (i.e., air pollutants, allergens and viruses) stimulate HBE cells to recruit effector T cells and trigger T helper 2 (Th2) cytokine secretion, which affects both HBE cells and HBSMCs via a feedback loop and further increases cytotoxicity in HBE cells and HBSMC contractility.^[^ ^10^ ^]^ However, the complex mechanism of asthma pathogenesis, especially the intercellular communication between HBE cells and HBSMCs, is incompletely elucidated. Hence, an exploration of comprehensive mechanisms contributing to PM_2.5_‐induced childhood asthma is urgently needed.

Recently, extracellular vesicles (EVs) have been regarded as important messengers in intercellular communication. EVs have been reported to be released by multiple types of cells under physiological or pathological conditions^[^ ^11^, ^12^ ^]^ and then transfer intracellular cargos (i.e., nucleic acids, lipidsand proteins) from the parent cells to recipient cells, thereby modulating the biological functions of the recipient cells.^[^ ^13^, ^14^ ^]^ Regarding the abovementioned EV‐packaged cargos, emerging studies have noted that microRNAs (miRNAs) are considered novel tools to investigate the pathologic mechanisms and biomarkers of asthma.^[^ ^15^ ^]^ Moreover, PM_2.5_ exposure regulates miRNA expression profiles in airway cells.^[^ ^16^, ^17^ ^]^ Based on these data, EV‐packaged miRNAs may function as important extracellular messengers in the PM_2.5_‐stimulated airway microenvironment. However, the role of EV‐packaged miRNAs in PM_2.5_‐induced childhood asthma remains unknown.

In the present study, we found that PM_2.5_‐treated HBE cells released EVs containing aberrantly expressed let‐7i‐5p that were taken up by normal HBE cells in the “horizontal” orientation and sensitive HBSMCs in the “longitudinal” orientation, leading to cytotoxicity and airway hyperresponsiveness, respectively. Accumulating studies have reported a role for let‐7i‐5p in multiple diseases.^[^ ^18^, ^19^, ^20^ ^]^ For example, the low expression of let‐7i‐5p caused by histone deacetylase 6 (HDAC6) directly increased expression levels of the target gene *TSP1* levels, thereby suppressing antiphagocytic and neoplastic behaviors of hepatocellular carcinoma.^[^ ^21^ ^]^ However, the biological function of let‐7i‐5p in PM_2.5_‐induced childhood asthma is unclear. Our study highlights the intercellular communication mechanism of EV‐packaged let‐7i‐5p‐mediated asthma and identifies EV‐packaged let‐7i‐5p as a novel predictor of PM_2.5_‐induced asthma.

## 2 Results

### 2.1 EVs Secreted by PM_2.5_ Stimulation “Horizontally” Induce Cytotoxicity in HBE Cells

**Figure 1:**
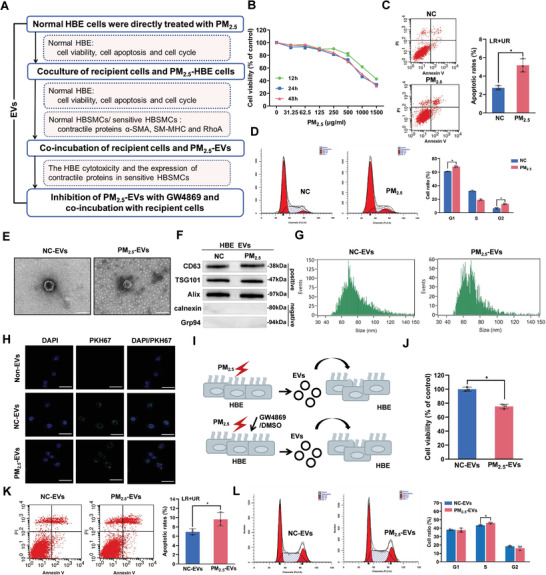
The effect of PM_2.5_‐EVs on cytotoxicity in HBE cells. PM_2.5_‐treated HBE cells were designated PM_2.5_‐HBE cells. EVs isolated from PM_2.5_‐treated HBE cells and NC‐treated HBE cells were designated PM_2.5_‐EVs and NC‐EVs, respectively. A) Flowchart of the strategy used to evaluate the effect of PM_2.5_‐EVs on cytotoxicity in HBE cells and the contractility of sensitive HBSMCs. B) The viability of HBE cells was evaluated using a CCK‐8 assay after PM_2.5_ treatment. C) The apoptosis of HBE cells was assessed using flow cytometry after PM_2.5_ treatment. D) The cell cycle of HBE cells was analyzed using flow cytometry after PM_2.5_ treatment. E) Purified PM_2.5_/NC‐EVs were identified using TEM. Scale bar, 100 nm. F) The EV‐specific markers (CD63, TSG101 and Alix) and negative controls (calnexin and Grp94) were evaluated using Western blotting. G) The size distributions of PM_2.5_/NC‐EVs were confirmed using NanoFCM. H) Representative fluorescence images of HBE cells after an incubation with PM_2.5_/NC‐EVs labelled with PKH67 (green) or non‐EVs. Scale bar, 25 µm. I) Schematic illustration of HBE cells incubated with PM_2.5_‐EVs or PM_2.5_‐EVs pretreated with DMSO/GW4869. J) The viability of HBE cells was evaluated using a CCK‐8 assay after an incubation with PM_2.5_/NC‐EVs. K) The apoptosis of HBE cells was assessed using flow cytometry after an incubation with PM_2.5_/NC‐EVs. L) The cell cycle of HBE cells was analyzed using flow cytometry after an incubation with PM_2.5_/NC‐EVs. Statistical significance was assessed using two‐tailed Student's *t*‐test. Values represent means ± SD. ^*^ *p* < 0.05.

The flowchart of the evaluation strategy for EVs is presented in **Figure** **1**A. First, we performed a direct PM_2.5_ exposure assay and found that PM_2.5_ dose‐dependently decreased the viability of HBE cells after exposure for different times (Table S1, Supporting Information). Compared with the PM_2.5_ group exposed for 12 h, PM_2.5_ concentrations greater than or equal to 250 µg mL^−1^ exhibited a significant difference in their ability to reduce the number of HBE cells after treatment for 24 or 48 h (Figure 1B). Because the concentration of PM_2.5_ may be up to 100–200 µg m^−3^ in winter, combined with children's daily inhalation of 10 m^3^ of air and the average volume of 6.2 mL of fluid in the lungs and airway, the corresponding PM_2.5_ concentration was calculated to be 161–323 µg mL^−1^.^[^ ^22^ ^]^ We selected 250 µg mL^−1^ PM_2.5_ and an exposure time of 24 h as the PM_2.5_ treatment conditions in this study to account for children exposed to high levels of PM_2.5_. As shown in Figure 1C,D, PM_2.5_ significantly induced apoptosis and G1/G2 arrest in normal HBE cells. Thus, PM_2.5_ exposure is cytotoxic to normal HBE cells.

Next, after coculture of normal and PM_2.5_/NC‐treated HBE cells in a Transwell plate for 24 h, the culture medium of PM_2.5_‐treated HBE cells exerted stronger cytotoxic effects than those observed on normal HBE cells exposed to NC‐treated HBE cells (Figure S1, Supporting Information), suggesting that PM_2.5_‐treated HBE cells may secrete EVs to induce cytotoxicity in HBE cells. Using visualization approaches, we found that EVs purified from the culture medium of PM_2.5_/NC‐treated HBE cells (PM_2.5_/NC‐EVs) exhibited a typical cup‐shaped morphology, were ≈100 nm in size, expressed the typical EV markers CD63, TSG101 and Alix, and lacked cellular contaminants such as calnexin and Grp94 (Figure 1E–G and Figure S2, Supporting Information). Fluorescence microscopy showed PKH67 green fluorescent dye in the cytoplasm of HBE cells, while no PKH67 green fluorescent dye was observed in the non‐EVs group, indicating that PM_2.5_/NC‐EVs were taken up by normal HBE cells (Figure 1H). However, this ability was abolished by inhibiting endocytosis with cytochalasin D (Figure S3, Supporting Information), indicating the efficient transportability of the secreted EVs. Subsequently, direct incubation with PM_2.5_‐EVs for 24 h markedly decreased HBE cell viability, promoted apoptosis, and stimulated S phase arrest (Figure 1I–L). Moreover, these aberrant cellular activities were rescued by the pharmacological inhibition of EV secretion with GW4869 (Figure 1I and Figure S4, Supporting Information). Taken together, these findings indicate that EVs secreted by PM_2.5_‐stimulated HBE cells are “horizontally” transported into normal HBE cells, with subsequent induction of cytotoxicity.

### 2.2 EVs Secreted Following PM_2.5_ Stimulation “Longitudinally” Promote the Contractility of Sensitive HBSMCs

**Figure 2:**
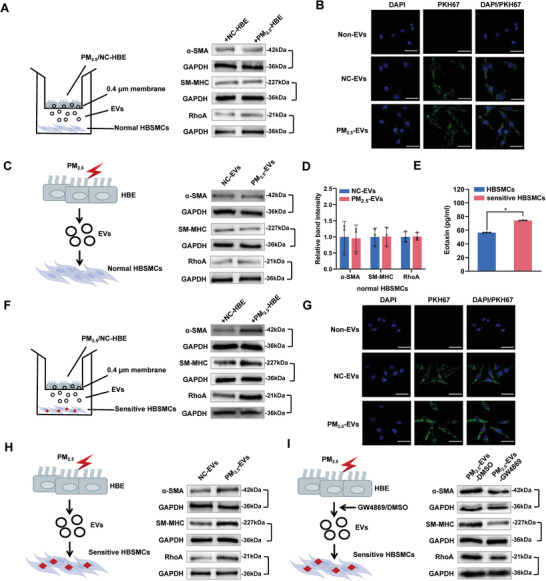
Effect of PM_2.5_‐EVs on the contractility of sensitive HBSMCs. The PM_2.5_/NC‐treated HBE cells are designated PM_2.5_/NC‐HBE cells. The red lightning signal represents PM_2.5_ exposure. A) The levels of the contractile proteins α‐SMA, SM‐MHC and RhoA in normal HBSMCs cocultured with PM_2.5_/NC‐HBE cells were analyzed using Western blotting. B) Representative fluorescence images of normal HBSMCs after an incubation with PM_2.5_/NC‐EVs labelled with PKH67 (green) or non‐EVs. Scale bar, 25 µm. C) The levels of the contractile proteins α‐SMA, SM‐MHC and RhoA in normal HBSMCs incubated with PM_2.5_/NC‐EVs were analyzed using Western blotting. D) The band intensity of contractile proteins in normal HBSMCs incubated with PM_2.5_/NC‐EVs. E) Normal HBSMCs were treated with IL‐13 to establish a sensitive HBSMC model. The expression of eotaxin in normal and sensitive HBSMCs was measured using an enzyme‐linked immunosorbent assay (ELISA) kit. F) The levels of the contractile proteins α‐SMA, SM‐MHC and RhoA in sensitive HBSMCs cocultured with PM_2.5_/NC‐HBE cells were analyzed using Western blotting. G) Representative fluorescence images of sensitive HBSMCs after an incubation with PM_2.5_/NC‐EVs labelled with PKH67 (green) or non‐EVs. Scale bar, 25 µm. H) The contractile proteins α‐SMA, SM‐MHC and RhoA in sensitive HBSMCs incubated with PM_2.5_/NC‐EVs were analyzed using Western blotting. I) The contractile proteins α‐SMA, SM‐MHC and RhoA in sensitive HBSMCs incubated with PM_2.5_/NC‐EVs pretreated with DMSO/GW4869 were analyzed using Western blotting. Statistical significance was assessed using two‐tailed Student's *t*‐test. Values represent means ± SD. ^*^ *p* < 0.05.

As PM_2.5_‐EVs may be “longitudinally” transported into normal HBSMCs, we detected the contractility of these cells by evaluating the expression of three identified vital proteins that regulate HBSMC contractility: α‐smooth muscle actin (α‐SMA), smooth muscle myosin heavy chain 11 (SM‐MHC) and RhoA (Figure 1A). No differences in the expression of contractile proteins were observed in normal HBSMCs after coculture with PM_2.5_‐treated HBE cells (**Figure** **2**A and Figure S5A, Supporting Information). Although PKH67‐labelled PM_2.5_/NC‐EVs were transferred into normal HBSMCs (Figure 2B and Figure S6, Supporting Information), PM_2.5_‐EVs did not regulate the expression of contractile proteins (Figure 2C,D). Based on these data, PM_2.5_ exposure may function as an inducing factor rather than an initiating factor of asthma by causing airway hyperresponsiveness and subsequently inducing asthma attacks. To simulate the condition of HBSMCs during asthma remission, we established a sensitive HBSMC model by using 100 ng mL^−1^ IL‐13 and found that sensitive HBSMCs exhibited increased eotaxin expression (Figure 2E). Compared with NC‐treated HBE cells, sensitive HBSMCs cocultured with PM_2.5_‐treated HBE cells showed higher expression levels of contractile proteins (Figure 2F and Figure S5B, Supporting Information). Similarly, “longitudinal” recipient‐sensitive HBSMCs displayed efficient uptake of PKH67‐labelled PM_2.5_/NC‐EVs (Figure 2G, and Figure S7, Supporting Information) and exhibited increased expression of contractile proteins after treatment with PM_2.5_‐EVs (Figure 2H and Figure S5C, Supporting Information). In contrast, GW4869 treatment of PM_2.5_‐treated HBE cells reduced the expression levels of contractile proteins in “longitudinal” recipient‐sensitive HBSMCs (Figure 2I and Figure S5D, Supporting Information). Collectively, these results suggest that PM_2.5_‐EVs are also “longitudinally” delivered to sensitive HBSMCs, enhancing their contractility.

### 2.3 EV‐Packaged Let‐7i‐5p Correlates with PM_2.5_ Exposure in Children with Asthma

**Figure 3:**
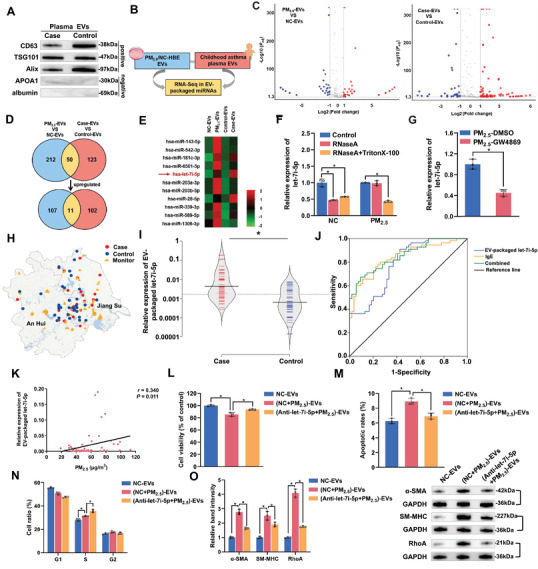
The role of EV‐packaged let‐7i‐5p in children with asthma and the phenotype of recipient cells. HBE cells were transfected with NC‐ or let‐7i‐5p inhibitor, exposed to NC/PM_2.5_ and then isolated the corresponding EVs, namely, NC‐EVs, (NC+PM_2.5_)‐EVs, and (Anti‐let‐7i‐5p+PM_2.5_)‐EVs, respectively. A) EV‐specific markers (CD63, TSG101 and Alix) and negative controls (APOA1 and albumin) were evaluated using Western blotting. B) Schematic model showing the generation of EV‐packaged miRNA expression profiles. C) Volcano plots of different EV‐packaged miRNAs in PM_2.5_‐treated HBE cell models and children with asthma. The red and blue plots indicate the differentially expressed EV‐packaged miRNAs. D) Venn diagram showing the overlapping upregulated EV‐packaged miRNAs. E) Hierarchical clustering analysis of the 11 overlapping upregulated EV‐packaged miRNAs identified from the Venn diagram. F) The level of let‐7i‐5p in the culture medium of PM_2.5_/NC‐treated HBE cells incubated with 2 µg mL^−1^ RNaseA alone or in combination with 0.1% Triton X‐100 was determined using RT‐qPCR. G) The level of let‐7i‐5p in the culture medium of PM_2.5_‐treated HBE cells exposed to DMSO or GW4869 was determined using RT‐qPCR. H) Locations of the individuals’ residences and air quality monitoring stations. I) The level of plasma EV‐packaged let‐7i‐5p was determined using RT‐qPCR. J) ROC curve analysis of plasma levels of EV‐packaged let‐7i‐5p and IgE. K) Correlation analysis was performed between plasma EV‐packaged let‐7i‐5p and PM_2.5_ exposure levels in children with asthma. L) The viability of HBE cells incubated with NC‐EVs, (NC+PM_2.5_)‐EVs or (Anti‐let‐7i‐5p+PM_2.5_)‐EVs was evaluated using a CCK‐8 assay. M) The apoptosis of HBE cells incubated with NC‐EVs, (NC+PM_2.5_)‐EVs or (Anti‐let‐7i‐5p+PM_2.5_)‐EVs was assessed using flow cytometry. N) The cell cycle of HBE cells incubated with NC‐EVs, (NC+PM_2.5_)‐EVs, or (Anti‐let‐7i‐5p+PM_2.5_)‐EVs was analyzed using flow cytometry. O) The levels of contractile proteins in sensitive HBSMCs incubated with NC‐EVs, (NC+PM_2.5_)‐EVs, or (Anti‐let‐7i‐5p+PM_2.5_)‐EVs were measured using Western blotting. Statistical significance was assessed using two‐tailed Student's *t*‐test. Values represent means ± SD. ^*^ *p* < 0.05.

Aiming to explore the epigenetic regulation of PM_2.5_‐induced childhood asthma, we verified EVs isolated from plasma in children (**Figure** **3**A and Figure S8, Supporting Information) and performed RNA sequencing on EV‐packaged miRNAs in plasma samples from children and PM_2.5_/NC‐treated HBE cells (Figure 3B). As shown in Figure 3C, 262 EV‐packaged miRNAs were differentially expressed in PM_2.5_‐EVs compared with NC‐EVs (118 upregulated and 144 downregulated; |fold change| >2, *P*_adj_ < 0.05), and 173 EV‐packaged miRNAs (113 upregulated and 60 downregulated; |fold change| >2, *P*_adj_ < 0.05) were differentially expressed in the comparison of plasma from children with asthma and healthy controls. Furthermore, we identified 11 overlapping upregulated EV‐packaged miRNAs in the cell models and plasma from children (Figure 3D), among which the most abundant EV‐packaged miRNA, let‐7i‐5p, was selected as the candidate miRNA for further verification (Figure 3E). Consistent with the RNA sequencing results, the level of EV‐packaged let‐7i‐5p was approximately two‐fold higher in PM_2.5_‐EVs than in NC‐EVs (Figure S9, Supporting Information). We confirmed the existing pattern of extracellular let‐7i‐5p by treating cells with RNaseA /Triton X‐100 and found that the level of let‐7i‐5p in the culture medium of PM_2.5_‐treated HBE cells did not change after RNaseA treatment but decreased markedly when RNaseA and Triton X‐100 were added simultaneously (Figure 3F). However, the let‐7i‐5p level was reduced in the culture medium of NC‐treated HBE cells after treatment with RNaseA. Additional EV inhibition experiments verified that the level of let‐7i‐5p in the culture medium of PM_2.5_‐treated HBE cells was significantly reduced after treatment with GW4869 (Figure 3G). Based on these results, extracellular let‐7i‐5p is mainly encapsulated in EVs instead of directly secreted.

Next, we sought to examine the association of EV‐packaged let‐7i‐5p with childhood asthma attacks and PM_2.5_ exposure based on 55 patients and 55 healthy controls (Figure 3H). The EV‐packaged let‐7i‐5p level remained stable after different freeze–thaw cycles (Figure S10A, Supporting Information) and room temperature incubation periods (Figure S10B, Supporting Information). Compared with the healthy controls (Table S2, Supporting Information), children with asthma had significantly higher plasma total IgE levels and peripheral blood eosinophil percentages (*p* < 0.001) and were exposed to higher levels of PM_2.5_. We further detected let‐7i‐5p levels in plasma EVs from children and found that the expression of EV‐packaged let‐7i‐5p was significantly increased in children with asthma (Figure 3I). Moreover, EV‐packaged let‐7i‐5p exhibited a high capacity to discriminate children with asthma from healthy controls, with an area under the curve (AUC) of 0.785 (95% confidence interval [CI] = 0.701–0.869; Figure 3J). Notably, the AUC of the combination of IgE and EV‐packaged let‐7i‐5p was increased to 0.855 (95% CI = 0.786–0.923), accompanied by increases in the sensitivity and specificity to 70.90% and 85.20%, respectively (Figure 3J). Correlation analysis revealed that the EV‐packaged let‐7i‐5p level exhibited a significant positive correlation with PM_2.5_ exposure in children with asthma but not in healthy controls (Figure 3K and Figure S11, Supporting Information). These observations imply that plasma levels of EV‐packaged let‐7i‐5p may be a biomarker for PM_2.5_‐induced asthma in children.

### 2.4 EV‐Packaged Let‐7i‐5p Affects Cytotoxicity in HBE Cells and the Contractility of Sensitive HBSMCs

We first transfected the let‐7i‐5p mimic into recipient cells to further determine the biological significance of EV‐packaged let‐7i‐5p in cytotoxicity in HBE cells and the contractility of sensitive HBSMCs and found that let‐7i‐5p overexpression reduced HBE cell growth, induced an increase in the apoptosis rate of HBE cells, and promoted S phase arrest (Figure S12A–D, Supporting Information). Moreover, let‐7i‐5p overexpression markedly increased the expression of contractile proteins in sensitive HBSMCs (Figure S12E,F, Supporting Information). Next, we cocultured HBE cells transfected with Cy3‐labelled let‐7i‐5p mimic with recipient cells and observed the red fluorescence of the labelled let‐7i‐5p in both “horizontal” recipient HBE cells and “longitudinal” recipient‐sensitive HBSMCs, indicating that EV‐packaged let‐7i‐5p was taken up by recipient cells (Figure S13A, Supporting Information). Subsequently, we evaluated the level of let‐7i‐5p after incubating the recipient cells with EVs and found that the cellular let‐7i‐5p levels were increased upon incubation with PM_2.5_‐EVs but not upon incubation with NC‐EVs or direct exposure to PM_2.5_ (Figure S13B,C, Supporting Information). These findings indicate that the enriched let‐7i‐5p in recipient cells is mainly transported by PM_2.5_‐EVs. We further examined the biological effect of EV‐packaged let‐7i‐5p on recipient HBE cells and sensitive HBSMCs. As shown in Figure 3L–O and Figure S14, Supporting Information, the effect of PM_2.5_‐EVs on enhancing cytotoxicity in HBE cells or increasing the contractility of sensitive HBSMCs was blocked when let‐7i‐5p within PM_2.5_‐treated HBE cells was antagonized by a let‐7i‐5p inhibitor. Moreover, the effects of PM_2.5_‐EVs on recipient cells were enhanced when let‐7i‐5p was overexpressed in recipient cells transfected with the let‐7i‐5p mimic (Figure S15, Supporting Information). Collectively, these data indicate that EV‐packaged let‐7i‐5p released by PM_2.5_‐treated HBE cells induces cytotoxicity in “horizontal” recipient HBE cells and increases the contractility of “longitudinal” recipient‐sensitive HBSMCs.

### 2.5 Let‐7i‐5p Is Packaged into EVs via ELAVL1 and Induces Asthma by Activating the MAPK Signaling Pathway

**Figure 4:**
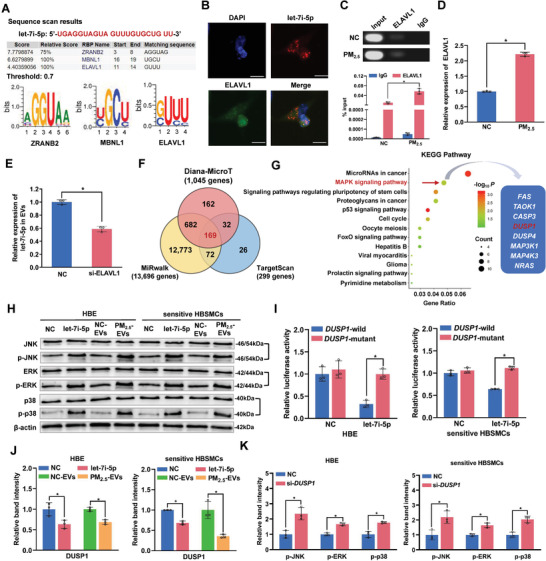
Let‐7i‐5p is packaged into PM_2.5_‐EVs in an ELAVL1‐dependent manner and regulates the *DUSP1* expression level in recipient cells. A) The specific interaction between the let‐7i‐5p sequence and RNA‐binding protein motifs was predicted by RBPDB (threshold value = 0.7). B) Immunofluorescence assessment of let‐7i‐5p (red) and ELAVL1 (green) colocalization in HBE cells. Scale bar, 20 µm. C) Upper panel: image of gel electrophoresis of PCR products from the RIP assay. Lower panel: an RIP assay with an anti‐ELAVL1 antibody or control IgG was performed on PM_2.5_/NC‐treated HBE cells. The level of let‐7i‐5p in the immunoprecipitates was determined using RT‐qPCR. D) The level of the ELAVL1 mRNA in PM_2.5_/NC‐treated HBE cells was determined using RT‐qPCR. E) The level of let‐7i‐5p in EVs derived from HBE cells transfected with si‐ELAVL1 was determined using RT‐qPCR. F) The potential target genes containing the let‐7i‐5p seed sequence were predicted using the Diana‐MicroT, MiRwalk and TargetScan databases. G) KEGG pathway enrichment analysis of the 169 target genes. H) The protein levels of MAPK pathway‐related signaling molecules in NC mimic‐, let‐7i‐5p mimic‐, NC‐EVs‐, or PM_2.5_‐EVs‐treated recipient cells (HBE cells and sensitive HBSMCs) were determined using Western blotting. I) The binding affinity of let‐7i‐5p for *DUSP1* was assessed using a dual‐luciferase reporter assay. J) The expression of the DUSP1 protein in NC mimic‐, let‐7i‐5p mimic‐, NC‐EVs, or PM_2.5_‐EVs‐treated HBE cells (left panel) and sensitive HBSMCs (right panel) was detected using Western blotting. K) The protein levels of MAPK pathway‐related signaling molecules in NC‐ or si‐*DUPS1‐*transfected HBE cells (left panel) and sensitive HBSMCs (right panel) were determined using Western blotting. Statistical significance was assessed using a two‐tailed Student's *t*‐test. Values represent means ± SD. ^*^ *p* < 0.05.

To investigate the mechanism by which let‐7i‐5p is loaded into EVs, we used the database of RNA‐binding protein specificities (RBPDB) to predict the specific interaction between let‐7i‐5p and the RNA‐binding proteins (RBPs) binding motif. As shown in **Figure** **4**A, three RBPs, including ELAV‐like protein 1 (ELAVL1), a well‐defined RBP for sorting miRNAs into EVs,^[^ ^23^, ^24^ ^]^ was predicted to specifically bind let‐7i‐5p (threshold 0.7). Notably, let‐7i‐5p FISH and ELAVL1 immunostaining indicated the colocalization of let‐7i‐5p and ELAVL1 in the cytoplasm of HBE cells (Figure 4B). Consistent with these findings, RNA binding protein immunoprecipitation (RIP) assays confirmed a more obvious interaction between let‐7i‐5p and ELAVL1 in PM_2.5_‐treated HBE cells than in NC‐treated HBE cells (Figure 4C), indicating that let‐7i‐5p is more likely to interact with ELAVL1 following exposure to PM_2.5_. Moreover, ELAVL1 was significantly upregulated in PM_2.5_‐treated HBE cells (Figure 4D), and the EV‐packaged let‐7i‐5p level was significantly reduced in cells with ELAVL1 knockdown (Figure 4E and Figure S16, Supporting Information). These findings suggest that ELAVL1 is responsible for the specific loading of let‐7i‐5p into EVs.

Next, we used three target prediction software programs and performed an overlap analysis to identify 169 putative targets of let‐7i‐5p (Figure 4F and Figure S17, Supporting Information). The subsequent Kyoto Encyclopedia of Genes and Genomes (KEGG) pathway enrichment analysis revealed consistent biological pathways involved in cellular activities (Figure 4G). Notably, the mitogen‐activated protein kinase (MAPK) signaling pathway was previously found to be functionally related to asthma, and among 8 candidate targets, dual‐specificity protein phosphatase 1 (DUSP1), a key inhibitor, directly inactivates Jun amino‐terminal kinase (JNK), extracellular signal‐regulated kinase (ERK), and p38 via dephosphorylation.^[^ ^25^ ^]^ Thus, we preferentially selected *DUSP1* as a putative downstream let‐7i‐5p target gene. As shown in Figure 4H and Figure S18, Supporting Information, let‐7i‐5p overexpression significantly increased the phosphorylation of JNK, ERK and p38 in recipient cells, similar to treatment with EV‐packaged let‐7i‐5p derived from PM_2.5_‐treated HBE cells. Further dual‐luciferase reporter assays showed that let‐7i‐5p significantly reduced the luciferase activity of the wild‐type *DUSP1* 3′‐UTR reporter (Figure 4I). Moreover, let‐7i‐5p overexpression or treatment with EV‐packaged let‐7i‐5p released by PM_2.5_‐treated HBE cells dramatically inhibited DUSP1 expression in both normal HBE cells and sensitive HBSMCs (Figure 4J and Figure S19, Supporting Information). Furthermore, *DUSP1* knockdown in recipient cells led to potent activation of the MAPK signaling pathway (Figure 4K and Figure S20, Supporting Information), induced cytotoxicity in HBE cells and increased the contractility of sensitive HBSMCs (Figure S21, Supporting Information). These findings imply that EV‐packaged let‐7i‐5p derived from PM_2.5_‐treated HBE cells may inhibit *DUSP1* expression to increase the phosphorylation of JNK, ERK and p38, thereby activating the MAPK signaling pathway and inducing asthma attacks through both “horizontal” and “longitudinal” mechanisms.

### 2.6 EV‐Packaged Let‐7i‐5p Mediates PM_2.5_ Exposure‐Induced Asthma In Vivo

**Figure 5:**
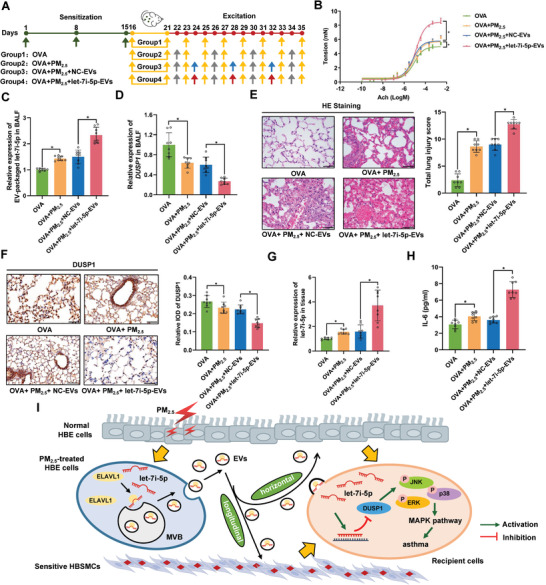
EV‐packaged let‐7i‐5p induces asthma in vivo. Thirty‐two female BALB/c mice were randomly assigned to the OVA group (group 1), OVA+PM_2.5_ group (group 2), OVA+PM_2.5_+NC‐EVs group (group 3), or OVA+PM_2.5_+let‐7i‐5p‐EVs group (group 4). A) Schematic showing the process used to establish the mouse model. The green arrows indicate that each mouse was injected with 28.8 µL of the OVA suspension containing 3.6 µg of OVA and 144 µg of Al(OH)_3_. The yellow arrows indicate that mice were exposed to 2% OVA for 20 min. The grey arrows indicate that mice were exposed to 1.57 mg kg^−1^ PM_2.5_. The blue arrows indicate that mice were challenged by an endotracheal instillation of 2 µg of NC‐EVs. The red arrows indicate that mice were challenged by an endotracheal instillation of 2 µg of let‐7i‐5p‐EVs. B) Detection of bronchoconstriction using myography. C) The level of EV‐packaged let‐7i‐5p in BALF was determined using RT‐qPCR. D) The level of *DUSP1* in BALF was determined using RT‐qPCR. E) Representative images of H&E staining in lung tissues and total lung injury scores for each group. Scale bar, 50 µm. F) Representative photographs and quantification of DUSP1 immunostaining in lung tissues. Scale bar, 50 µm. G) The level of let‐7i‐5p in lung tissues was determined using RT‐qPCR. H) The concentration of IL‐6 in BALF was measured using ELISA. I) In the proposed model, EV‐packaged let‐7i‐5p secreted by PM_2.5_‐treated HBE cells induce asthma by regulating the expression of its target gene *DUSP1* and activating the MAPK signaling pathway in recipient cells. MVB, multivesicular bodies. Statistical significance was assessed using two‐tailed Student's *t*‐test. Values represent means ± SD. ^*^ *p* < 0.05.

We established a model of ovalbumin (OVA)‐induced asthma in juvenile mice and subjected the mice to the indicated treatments to assess the role of EV‐packaged let‐7i‐5p in PM_2.5_‐induced asthma in vivo (**Figure** **5**A). Consistent with the in vitro observations, treatment with PM_2.5_ or PM_2.5_+let‐7i‐5p EVs significantly increased bronchoconstriction (Figure 5B), increased the levels of three contractile proteins (Figure S22, Supporting Information), upregulated EV‐packaged let‐7i‐5p in bronchoalveolar lavage fluid (BALF) (Figure 5C), reduced the *DUSP1* mRNA level in BALF (Figure 5D), and damaged lung tissues (Figure 5E); these effects were accompanied by DUSP1 downregulation (Figure 5F) and an elevated let‐7i‐5p level in lung tissues (Figure 5G). Compared with the OVA or OVA+PM_2.5_+NC‐EVs group, the levels of phosphorylated JNK, ERK and p38 were increased in the OVA+PM_2.5_ or OVA+PM_2.5_+let‐7i‐5p EVs group (Figure S23, Supporting Information), indicating that EV‐packaged let‐7i‐5p stimulated PM_2.5_‐induced asthma by activating the MAPK signaling pathway. Furthermore, the IL‐6 concentration in BALF was significantly increased by the same treatment, further confirming the establishment of the mouse model of OVA‐induced asthma (Figure 5H). The observations described the above highlight that EV‐packaged let‐7i‐5p promotes PM_2.5_‐induced airway contraction and cytotoxicity by inhibiting its target gene *DUSP1*.

## 3 Discussion

In the current study, we first conducted RNA sequencing of EV‐packaged miRNAs in plasma from children with asthma and healthy controls and PM_2.5_/NC‐treated HBE cell models, and we identified a novel miRNA, let‐7i‐5p, expressed at high levels in PM_2.5_‐EVs and plasma from children with asthma. Let‐7i‐5p was packaged into EVs through an interaction with ELAVL1 and then taken up by “horizontal” recipient normal HBE cells and “longitudinal” recipient‐sensitive HBSMCs. Functionally, EV‐packaged let‐7i‐5p activated the MAPK signaling pathway by inhibiting its target gene *DUSP1*, resulting in cytotoxicity in “horizontal” recipient HBE cells and increased contractility of “longitudinal” recipient‐sensitive HBSMCs (Figure 5I).

Because the composition of PM_2.5_ is easily affected by the geographical location and meteorological conditions,^[^ ^26^, ^27^ ^]^ we used National Institute of Standards and Technology (NIST) reference materials that have a well‐characterized chemical content of PM_2.5_ and represent urban PM_2.5_ levels. Our results revealed that PM_2.5_ decreased the viability of normal HBE cells, consistent with our previous studies.^[^ ^28^ ^]^ We then hypothesized that PM_2.5_‐treated HBE cells might secrete EVs to regulate the biological function of neighboring or distant cells. Our coculture experiments indicated that EVs released by PM_2.5_‐treated HBE cells may induce cytotoxicity in normal HBE cells. As expected, PM_2.5_‐EVs were efficiently transferred into recipient HBE cells^[^ ^29^, ^30^ ^]^ and then “horizontally” induce cytotoxicity in HBE cells; however, this effect was abolished by pretreatment of recipient HBE cells with the EV secretion inhibitor GW4869. The biological effects of PM_2.5_ and PM_2.5_‐EVs on the cell cycle of HBE cells were not consistent because PM_2.5_‐EVs represent the effect of aberrantly expressed intracellular cargos carried by PM_2.5_‐EVs. Similarly, PM_2.5_‐EVs were taken up by recipient‐sensitive HBSMCs and subsequently promoted sensitive HBSMC contractility by directly increasing the expression of α‐SMA, SM‐MHC and RhoA. Thus, PM_2.5_‐EVs play a key role in PM_2.5_‐induced asthma through both “horizontal” and “longitudinal” mechanisms. The air–liquid interface (ALI) model is becoming an efficient and realistic tool for bronchial epithelial toxicity testing following exposure to air pollutants.^[^ ^31^ ^]^ A better understanding of the biological function of EV‐packaged let‐7i‐5p in PM_2.5_‐induced asthma based on the ALI model is needed.

Based on RNA sequencing, we determined that let‐7i‐5p was overexpressed in PM_2.5_‐EVs and plasma‐derived EVs from children with asthma and was mainly encapsulated in EVs instead of being freely secreted. Let‐7i‐5p, a member of the lethal‐7 (let‐7) miRNA family, has been detected in various tissues and cell types (i.e., lung tissue, smooth muscle, epithelial cells, and macrophages).^[^ ^15^, ^32^ ^]^ To date, only one study has reported that let‐7i‐5p was differentially expressed in bronchial cells from steroid‐naive subjects with asthma and was downregulated in patients with asthma,^[^ ^25^ ^]^ which is inconsistent with our results. The main explanation for the inconsistency might be the difference in the ages and ethnicity of the subjects. Various studies have indicated that EVs are ideal biomarkers of many diseases; in addition to being stable in body fluids, EVs carry disease‐specific functional molecules.^[^ ^33^ ^]^ The advantages of EV‐packaged let‐7i‐5p are based on three aspects. First, we determined that plasma EV‐packaged let‐7i‐5p levels were positively correlated with PM_2.5_ exposure in children with asthma, indicating that let‐7i‐5p is a specific predictor of PM_2.5_‐induced asthma in children. Second, we observed a significant increase in EV‐packaged let‐7i‐5p levels produced by PM_2.5_‐stimulated HBE cells, and EV‐packaged let‐7i‐5p performs crucial functions in effector cells of asthma (HBE and sensitive HBSMCs), which is helpful for both explaining the biological mechanism of PM_2.5_‐induced asthma and further investigating therapeutic approaches. Third, EV‐packaged let‐7i‐5p was stable in plasma and had the ability to discriminate children with asthma from healthy controls with acceptable sensitivity and specificity. These observations imply that EV‐packaged let‐7i‐5p has the potential to serve as a specific biomarker for PM_2.5_‐induced childhood asthma compared with a series of classical predictors. Furthermore, further investigations are needed to determine whether EV‐packaged let‐7i‐5p is related to asthma induced by other allergens.

Interestingly, we revealed that EV‐packaged let‐7i‐5p was delivered into recipient cells and then “horizontally” induced cytotoxicity in HBE cells and “longitudinally” promoted the contractility of sensitive HBSMCs. This conclusion was based on the findings listed below. 1) Let‐7i‐5p overexpression caused cytotoxicity in HBE cells and increased the expression of contractile proteins in sensitive HBSMCs; 2) HBE cells transfected with Cy3‐labelled let‐7i‐5p mimic were cocultured with recipient cells^[^ ^34^ ^]^ to show that EV‐packaged let‐7i‐5p produced by HBE cells was directly transferred into either “horizontal” or “longitudinal” recipient cells; 3) Let‐7i‐5p overexpression in recipient cells was caused mainly by treatment with PM_2.5_‐EVs rather than by direct exposure to PM_2.5_; 4) Incubation with (Anti‐let‐7i‐5p+PM_2.5_)‐EVs significantly decreased the capacity of PM_2.5_‐EVs to inhibit cytotoxicity in “horizontal” recipient HBE cells and decrease α‐SMA, SM‐MHC and RhoA expression levels in “longitudinal” recipient‐sensitive HBSMCs; and 5) Transfection of the let‐7i‐5p mimic into recipient cells significantly increased the capacity of PM_2.5_‐EVs to induce asthma. Asthma is driven by a complex cellular communication network composed of multiple cell types.^[^ ^10^, ^35^ ^]^ The cell–cell communication of EV‐packaged let‐7i‐5p with other cell types in the cell microenvironment of asthma must be investigated in further studies. Moreover, PM_2.5_ accelerated the production of EV‐packaged let‐7i‐5p by HBE cells by upregulating levels of the EV packaging‐related protein ELAVL1 rather than increasing cellular let‐7i‐5p expression levels. Notably, previous studies have reported that EV‐packaged miRNAs are selectively packaged into EVs by several RBPs, including hnRNPA2B1, hnRNPQ and ELAVL1.^[^ ^23^, ^36^, ^37^ ^]^ Consistent with these results, ELAVL1 (also called HuR) specifically bound let‐7i‐5p through a specific motif (GUUU) and directed the sorting of let‐7i‐5p into EVs in the present study, which might suggest unique strategies to eliminate EV‐packaged let‐7i‐5p during PM_2.5_ exposure. In addition to RBPs, Ago2 and Y‐box protein 1 have been reported to participate in the EV packaged export of miRNA.^[^ ^38^, ^39^ ^]^ Additionally, lncRNA–miRNA interactions have recently been described as a new regulatory mechanism for EV‐packaged miRNA export.^[^ ^40^ ^]^ Thus, we need to fully elucidate the regulatory mechanism in further studies.

The MAPK signaling pathway is involved in almost all aspects of asthma pathophysiology, and its activity is self‐adjusting between phosphorylation and dephosphorylation.^[^ ^41^, ^42^ ^]^ Extracellular stimulation (by environmental exposure, growth factors or proinflammatory cytokines) leads mainly to the activation of the three main MAPK signaling pathway modules: JNK, ERK and p38.^[^ ^43^ ^]^ Here, we found that EV‐packaged let‐7i‐5p secreted by PM_2.5_‐treated HBE cells increased the levels of phosphorylated JNK, ERK and p38 by suppressing the expression of the let‐7i‐5p target gene *DUSP1* in both HBE cells and sensitive HBSMCs, thereby activating the MAPK signaling pathway to induce asthma. Consistent with our in vitro results, juvenile mice treated with EV‐packaged let‐7i‐5p exhibited more severe bronchial contraction, higher levels of EV‐packaged let‐7i‐5p in BALF and lower DUSP1 protein levels in lung tissues compared to those in control mice (OVA group or OVA+PM_2.5_+NC‐EVs group). Because all the functions we investigated were based on OVA‐induced asthmatic mouse models treated with PM_2.5_, OVA and PM_2.5_ could be used as internal controls for the OVA+PM_2.5_+NC/let‐7i‐5p EVs group, thereby eliminating the effect of OVA or PM_2.5_ on target molecules and other indices. Interestingly, EV‐packaged let‐7i‐5p regulated the MAPK signaling pathway through the target gene *DUSP1* and increased IL‐6 expression levels. These results indicate that EV‐packaged let‐7i‐5p may regulate the release of inflammatory factors. Thus, studies exploring the function of let‐7i‐5p in inflammatory cells are urgently needed. Due to the lower amount of plasma in the juvenile mouse model, we were unable to detect plasma EV‐packaged let‐7i‐5p in vivo. We therefore isolated BALF samples in vivo to measure the expression of EV‐packaged let‐7i‐5p. Overexpression and knockout of let‐7i‐5p in mice exposed to OVA and PM_2.5_ are necessary controls to further verify the biological effects of EV‐packaged let‐7i‐5p on PM_2.5_‐induced asthma. Moreover, the CRISPR/Cas9 system will be used to generate let‐7i‐5p knockout cell lines, thereby further validating the effect of EV‐packaged let‐7i‐5p on PM_2.5_‐induced asthma in vivo and in vitro. House dust mites (HDMs), important natural allergens, have been shown to more closely mimic human asthma than OVA when constructing an asthmatic mouse model.^[^ ^44^ ^]^ Therefore, we will use the HDM‐induced asthmatic mouse model to further validate our results.

In summary, our findings provide evidence of a mechanism underlying PM_2.5_‐induced childhood asthma in which EV‐packaged let‐7i‐5p “horizontally” induces cytotoxicity in HBE cells and “longitudinally” promotes the contractility of sensitive HBSMCs by inhibiting the expression of its target gene *DUSP1*. In addition, EV‐packaged let‐7i‐5p was upregulated in the plasma of children with asthma, and the EV‐packaged let‐7i‐5p level was positively correlated with PM_2.5_ exposure. Our results not only revealed a crucial mechanism of EV‐packaged miRNA‐regulated cell–cell communication from PM_2.5_‐treated HBE cells to recipient cells to induce asthma, but also opened a new avenue for a diagnostic strategy and therapeutic approach for asthma in children exposed to high levels of PM_2.5._

## 4 Experimental Section

### Study Subjects and Clinical Samples

Beginning in July 2017, the authors recruited 110 children from Children's Hospital of Nanjing Medical University (Nanjing, China) and collected their plasma samples. Children with asthma were diagnosed according to the guidelines for the diagnosis, prevention and treatment of childhood asthma (2016 edition) formulated by the Chinese Medical Association. During the same period, non‐asthmatic children who were age‐ and sex‐matched by the frequency with the cases were selected as healthy controls. Written informed consent forms were signed by the children and their parents, and the study protocol was approved by the Ethics Committee of Nanjing Medical University (2017‐392).

### PM_2.5_ Exposure Assessment

The PM_2.5_ concentration distribution was obtained from China National Environmental Monitoring Center (CNEMC) data provided by Qingyue Data (https://data.epmap.org). The PM_2.5_ concentration at the site 30 days before recruitment of the subjects into this study was recorded as the average concentration in the case‐control study. XGeocoding software was utilized to obtain the latitude and longitude of the subjects’ residence and monitoring sites. Inverse distance weighted interpolation (IDW) is an important spatial interpolation method in ArcGIS software (version 10.2), that was employed to evaluate the individual PM_2.5_ exposure concentrations.^[^ ^45^, ^46^ ^]^ The details are explained as follows:

(1) $$Z\left(u\right)\;=\;\frac{\sum_{i\;=\;1}^{n}\frac{1}{d^{a}}\;z\left(u_{i}\right)}{\sum_{i\;=\;1}^{n}\frac{1}{d^{a}}}$$

in Equation (1), *Z* (*u*) denotes the PM_2.5_ exposure concentration of subjects, *z*(*u*_i_) is the observed value at the *n* measured sites, and *d^a^* represents the inverse distance weighted interpolation (IDW) weight distance.

### Treatment of Recipient Cells with PM_2.5_

Standard reference material 2786 (SRM2786) from the NIST was used as the PM_2.5_ reference in this study.^[^ ^28^ ^]^ The PM_2.5_ standard was suspended in culture medium at a concentration of 2 mg mL^−1^. When recipient cells reached 50–60% confluence at the bottom of the petri dish, recipient cells were treated with different concentrations (0, 31.25, 62.5, 125, 250, 500, 1000, or 1500 µg mL^−1^) of the stock PM_2.5_ standard suspension for 12, 24 or 48 h.^[^ ^47^ ^]^

### EV Isolation, Characterization, and Internalization

EVs were isolated from the culture medium of HBE cells with an ExoQuick TC Kit (SBI, USA), and EVs were purified from the plasma of children using an ExoQuick Plasma Prep with Thrombin Kit (SBI, USA). The shape and size of EVs were assessed using transmission electron microscopy (TEM) (Tecnai G2, FEI, USA). The size distribution of EVs was quantified using nano‐flow cytometry (NanoFCM) with a U30 Flow NanoAnalyzer (NanoFCM, Inc., China) and technical assistance provided by KeyGEN Biotech Co. Ltd (Jiangsu Province, China). Western blotting was utilized to detect the EV protein markers CD63 (antibody: ab134045, Abcam, USA), TSG101 (antibody: ab125011, Abcam, USA) and Alix (antibody: 92880, CST, USA), and the negative controls calnexin (antibody: ab133615, Abcam, USA), Grp94 (antibody: ab108606, Abcam, USA), APOA1 (antibody: ab52945, Abcam, USA), and albumin (antibody: ab207327, Abcam, USA) to confirm that active substances isolated from culture medium or plasma were EVs. The 2 µg of PM_2.5_/NC‐EVs diluted with 200 µL of PBS were extracted (100 µL) and then labelled with 4 µL of PKH67 (green fluorescence; Sigma, USA) and 1 mL Diluent C (Sigma, USA). After an incubation for 4 min at room temperature, PM_2.5_/NC‐EV labelling was terminated by the addition of 2 mL of 0.5% bovine serum albumin (BSA). Dyed EVs were isolated from the above staining liquid using an ExoQuick TC Kit to remove unbound dye from the PKH67‐labelled PM_2.5_/NC‐EVs. The dyed EVs were added to recipient cells and incubated for 3 h. Fluorescence microscopy (Zeiss, Germany) was used to visualize PKH67‐labelled EVs in recipient cells. The details of this protocol are described in the authors' previous study.^[^ ^30^ ^]^

### EV Treatment

The concentration of EVs was determined with a bicinchoninic acid (BCA) protein assay kit (Beyotime Institute of Biotechnology, China). For in vitro treatment, 2 µg of EVs (equivalent to ≈1 × 10^7^ of the indicated HBE cells) were incubated with 2 × 10^5^ recipient cells for 24 h. EV secretion was inhibited by pretreating normal HBE cells with 20 µm GW4869 (Sigma, USA) or 0.005% DMSO (Sigma, USA) as a control for 6 h and then exposing cells to the PM_2.5_ standard for 24 h. The culture medium of these cells was prepared for EV isolation or RNA extraction. The indicated HBE cells were treated with 2 µg mL^−1^ RNaseA (Beyotime Institute of Biotechnology, China) alone or in combination with 0.1% Triton X‐100 (Beyotime Institute of Biotechnology, China) for 30 min, and the expression of miRNAs in the culture medium of HBE cells was detected using real‐time fluorescence quantitative polymerase chain reaction (RT‐qPCR) to investigate the expression pattern of extracellular miRNAs.

### Analysis of the EV‐Packaged miRNA Expression Profile

EV‐packaged RNA was isolated from the plasma of children with asthma or non‐asthmatic children, from which two pooled plasma samples of 4.8 mL each (4.8 mL = 8 samples × 600 µL/sample) were obtained (Table S3, Supporting Information). Moreover, EV‐packaged RNA was isolated from the culture medium of PM_2.5_/NC‐treated HBE cells. The EV‐packaged miRNA expression profiles of plasma from children and culture medium from the cell models were generated with the Illumina HiSeq 2500 platform (Novogene Co., Ltd., China).

### Western Blot Analysis

The indicated cells or EV‐packaged lysates were obtained using RIPA lysis buffer mixed with 0.5% PMSF and centrifuged at 15, 000 rpm for 15 min at 4 °C. Protein concentrations were determined using the BCA kit. Equal amounts of protein (60 µg) were separated by sodium dodecyl sulfate–polyacrylamide gel electrophoresis (SDS‐PAGE) and transferred to polyvinylidene fluoride (PVDF) membranes. Subsequently, membranes were incubated overnight at 4 °C with anti‐α‐SMA (19 245, CST, USA), anti‐SM‐MHC (ab53219, Abcam, USA), anti‐RhoA (2117, CST, USA), anti‐ELAVL1 (ab200342, Abcam, USA), anti‐DUSP1 (2 455 724, Millipore, USA), anti‐MAPK family (9926, CST, USA), or anti‐phospho‐MAPK family (9910, CST, USA) antibodies. Glyceraldehyde phosphate dehydrogenase (GAPDH) and *β*‐actin were used as loading controls for normalization. Immunoreactive proteins were examined using a Bio‐Rad gel imaging system (Bio‐Rad, USA).

### Establishment of the Juvenile Mouse Model of Asthma

Thirty‐two juvenile BALB/c mice (3 weeks old, female) were randomly divided into four groups of eight mice per group: the OVA group (OVA), OVA+PM_2.5_ exposure group (OVA+PM_2.5_), OVA+PM_2.5_ exposure+NC‐EVs injection group (OVA+PM_2.5_+NC‐EVs), and OVA+PM_2.5_ exposure+let‐7i‐5p‐EVs injection group (OVA+PM_2.5_+let‐7i‐5p‐EVs). On days 1, 8 and 15, juvenile mice in the four groups were intraperitoneally injected with 0.2 mL of the OVA suspension containing 25 µg of OVA (Sigma, USA) and 1 mg of Al(OH)_3_ (Aladdin, China). All mice were then exposed to nebulized 2% OVA on days 16–21, 23, 25, 27, 29, 31, 33, and 35 (20 min each time). Juvenile mice in all groups except the OVA group were exposed to 1.57 mg kg^−1^ nebulized PM_2.5_ on days 22, 26, 30, and 34. Additionally, mice in the OVA+PM_2.5_+NC/let‐7i‐5p‐EVs group received NC/let‐7i‐5p EVs (2 µg/sample) via intraperitoneal injection on days 24, 28 and 32. All animal studies were approved by the Institutional Animal Care and Use Committee of Nanjing Medical University (IACUC‐2004036).

### Statistical Analysis

Significant differences between the two groups were analyzed using Student's *t*‐test or Pearson's *χ* ^2^ test. The ROC curve was analyzed to obtain the AUC values and evaluate the capability for discriminating cases from healthy controls. Spearman's correlation analysis was utilized to determine the relationship between PM_2.5_ exposure and the plasma level of EV‐packaged let‐7i‐5p. Three target prediction software programs, Diana‐MicroT, MiRwalk and TargetScan, were used to predict putative targets of let‐7i‐5p. KEGG pathway analyses were performed with Database for Annotation, Visualization and Integrated Discovery (DAVID) 6.8 software (https://david‐d.ncifcrf.gov/) to identify the main biological pathways of the differentially expressed genes. RBPDB(http://rbpdb.ccbr.utoronto.ca/) was used to predict the interaction between motifs of RBPs and let‐7i‐5p^.[^ ^48^ ^]^ *p* < 0.05 was considered statistically significant. All experiments were replicated at least three times. Quantitative data are presented as the means ± standard deviations (SD). Statistical analyses were performed with SAS 9.1.3 software (SAS Institute, Inc., USA). Additional experimental methods are described in the Supporting Information Materials and Methods.

## Author's Contributions

R.Z., M.D., and M.T. contributed equally to this work. Z.Z., M.W., and M.D. designed and supervised the study. M.T., R.Z., C.G., R.X., and H.C. contributed to collect the characteristics of subjects and recruit study subjects. R.Z., C.W., J.L., F.G., and Z.M. contributed to the functional experiments. R.Z., M.D., and Z.Z. prepared the manuscript.

## Conflict of Interest

The authors declare no conflict of interest.

## Supporting information

Supporting Information

## Data Availability

The data that support the findings of this study are available from the corresponding author upon reasonable request.

## References

[1] P. W. Hekking , R. R. Wener , M. Amelink , A. H. Zwinderman , M. L. Bouvy , E. H. Bel , J. Allergy Clin. Immunol. 2015, 135, 896. 25441637 10.1016/j.jaci.2014.08.042

[2] H. W. Hallas , B. L. Chawes , M. A. Rasmussen , L. Arianto , J. Stokholm , K. Bønnelykke , H. Bisgaard , PLoS Med. 2019, 16, e1002722. 30620743 10.1371/journal.pmed.1002722 PMC6324782

[3] Lancet Respir. Med. 2017, 5, 691.28822787

[4] Lancet 2018, 392, 1736.30496103

[5] A. Papi , C. Brightling , S. E. Pedersen , H. K. Reddel , Lancet 2018, 391, 783. 29273246 10.1016/S0140-6736(17)33311-1

[6] F. Chen , Z. Lin , R. Chen , D. Norback , C. Liu , H. Kan , Q. Deng , C. Huang , Y. Hu , Z. Zou , W. Liu , J. Wang , C. Lu , H. Qian , X. Yang , X. Zhang , F. Qu , J. Sundell , Y. Zhang , B. Li , Y. Sun , Z. Zhao , Environ. Pollut. 2018, 232, 329. 28970023 10.1016/j.envpol.2017.08.072

[7] E. Garcia , K. T. Berhane , T. Islam , R. Mcconnell , R. Urman , Z. Chen , F. D. Gilliland , JAMA, J. Am. Med. Assoc. 2019, 321, 1906. 10.1001/jama.2019.5357 PMC6537847 31112259

[8] W. Gao , L. Li , Y. Wang , S. Zhang , I. M. Adcock , P. J. Barnes , M. Huang , X. Yao , Respirology 2015, 20, 722. 25868842 10.1111/resp.12542

[9] I. Bara , A. Ozier , J.‐M. T. De Lara , R. Marthan , P. Berger , Eur. Respir. J. 2010, 36, 1174. 21037369 10.1183/09031936.00019810

[10] D. J. Erle , D. Sheppard , J. Cell Biol. 2014, 205, 621. 24914235 10.1083/jcb.201401050 PMC4050726

[11] A. A. Farooqi , N. N. Desai , M. Z. Qureshi , D. R. N. Librelotto , M. L. Gasparri , A. Bishayee , S. M. Nabavi , V. Curti , M. Daglia , Biotechnol. Adv. 2018, 36, 328. 29248680 10.1016/j.biotechadv.2017.12.010

[12] R. Xu , D. W. Greening , H.‐J. Zhu , N. Takahashi , R. J. Simpson , J. Clin. Invest. 2016, 126, 1152. 27035807 10.1172/JCI81129 PMC4811150

[13] D. M. Pegtel , S. J. Gould , Annu. Rev. Biochem. 2019, 88, 487. 31220978 10.1146/annurev-biochem-013118-111902

[14] R. Kalluri , V. S. Lebleu , Science 2020, 367, eaau6977. 32029601 10.1126/science.aau6977 PMC7717626

[15] B. Sastre , J. A. Cañas , J. M. Rodrigo‐Muñoz , V. Del Pozo , Front. Immunol. 2017, 8, 826. 28785260 10.3389/fimmu.2017.00826 PMC5519536

[16] J. Li , Q. Zhou , Y. Liang , W. Pan , Y. Bei , Y. Zhang , J. Wang , Z. Jiao , Ann. Transl. Med. 2018, 6, 209. 30023372 10.21037/atm.2018.06.09 PMC6035978

[17] L. Song , D. Li , X. Li , L. Ma , X. Bai , Z. Wen , X. Zhang , D. Chen , L. Peng , Environ. Toxicol. Pharmacol. 2017, 50, 192. 28192748 10.1016/j.etap.2017.02.011

[18] Y. Hu , G. Jin , B. Li , Y. Chen , L. Zhong , G. Chen , X. Chen , J. Zhong , W. Liao , Y. Liao , Y. Wang , J. Bin , Clin. Sci. 2019, 133, 425. 10.1042/CS20181002 30679264

[19] J. Jin , F. Qian , D. Zheng , W. He , J. Gong , Q. He , Int. J. Nanomed. 2021, 16, 3565. 10.2147/IJN.S299969 PMC8164705 34079249

[20] Z. Shi , S. K. Y. To , S. Zhang , S. Deng , M. Artemenko , M. Zhang , J. Tang , J.‐Z. Zeng , A. S. T. Wong , Theranostics 2021, 11, 3376. 33537093 10.7150/thno.52190 PMC7847671

[21] H. D. Yang , H. S. Kim , S. Y. Kim , M. J. Na , G. Yang , J. W. Eun , H. J. Wang , J. Y. Cheong , W. S. Park , S. W. Nam , Hepatology 2019, 70, 1262. 30991448 10.1002/hep.30657

[22] X. Ding , M. Wang , H. Chu , M. Chu , T. Na , Y. Wen , D. Wu , B. Han , Z. Bai , W. Chen , J. Yuan , T. Wu , Z. Hu , Z. Zhang , H. Shen , Toxicol. Lett. 2014, 228, 25. 24769257 10.1016/j.toxlet.2014.04.010

[23] Z. Li , X. Zhou , M. Wei , X. Gao , L. Zhao , R. Shi , W. Sun , Y. Duan , G. Yang , L. Yuan , Nano Lett. 2019, 19, 19. 30517011 10.1021/acs.nanolett.8b02689

[24] Y. Shi , Z. Wang , X. Zhu , L. Chen , Y. Ma , J. Wang , X. Yang , Z. Liu , Int. J. Clin. Oncol. 2020, 25, 89. 31506750 10.1007/s10147-019-01532-9

[25] O. D. Solberg , E. J. Ostrin , M. I. Love , J. C. Peng , N. R. Bhakta , L. Hou , C. Nguyen , M. Solon , C. Nguyen , A. J. Barczak , L. T. Zlock , D. P. Blagev , W. E. Finkbeiner , K. M. Ansel , J. R. Arron , D. J. Erle , P. G. Woodruff , Am J. Respir. Crit. Care Med. 2012, 186, 965. 22955319 10.1164/rccm.201201-0027OC PMC3530212

[26] K. Sawyer , S. Mundandhara , A. J. Ghio , M. C. Madden , J. Toxicol. Environ. Health, Part A 2010, 73, 41. 10.1080/15287390903248901 19953419

[27] S. Becker , L. A. Dailey , J. M. Soukup , S. C. Grambow , R. B. Devlin , Y‐C. T. Huang , Environ. Health Perspect. 2005, 113, 1032. 16079075 10.1289/ehp.7996 PMC1280345

[28] Q. Yuan , H. Zhu , H. Liu , M. Wang , H. Chu , Z. Zhang , J. Hazard. Mater. 2021, 415, 125573. 33730643 10.1016/j.jhazmat.2021.125573

[29] Y. Liu , F. Luo , B. Wang , H. Li , Y. Xu , X. Liu , L. Shi , X. Lu , W. Xu , L. Lu , Y. Qin , Q. Xiang , Q. Liu , Cancer Lett. 2016, 370, 125. 26525579 10.1016/j.canlet.2015.10.011

[30] R. Zheng , M. Du , X. Wang , W. Xu , J. Liang , W. Wang , Q. Lv , C. Qin , H. Chu , M. Wang , L. Yuan , J. Qian , Z. Zhang , Mol. Cancer 2018, 17, 143. 30285771 10.1186/s12943-018-0880-3 PMC6169076

[31] S. Upadhyay , L. Palmberg , Toxicol. Sci. 2018, 164, 21. 29534242 10.1093/toxsci/kfy053

[32] H. Lee , S. Han , C. S. Kwon , D. Lee , Protein Cell 2016, 7, 100. 26399619 10.1007/s13238-015-0212-y PMC4742387

[33] S. Stremersch , S. C. De Smedt , K. Raemdonck , J. Controlled Release 2016, 244, 167. 10.1016/j.jconrel.2016.07.054 27491882

[34] W. Ying , M. Riopel , G. Bandyopadhyay , Y. Dong , A. Birmingham , J. B. Seo , J. M. Ofrecio , J. Wollam , A. Hernandez‐Carretero , W. Fu , P. Li , J. M. Olefsky , Cell 2017, 171, 372. 28942920 10.1016/j.cell.2017.08.035

[35] J. T. Olin , M. E. Wechsler , BMJ 2014, 349, g5517. 25420994 10.1136/bmj.g5517

[36] C. Villarroya‐Beltri , C. Gutiérrez‐Vázquez , F. Sánchez‐Cabo , D. Pérez‐Hernández , J. Vázquez , N. Martin‐Cofreces , D. J. Martinez‐Herrera , A. Pascual‐Montano , M. Mittelbrunn , F. Sánchez‐Madrid , Nat. Commun. 2013, 4, 2980. 24356509 10.1038/ncomms3980 PMC3905700

[37] L. Santangelo , G. Giurato , C. Cicchini , C. Montaldo , C. Mancone , R. Tarallo , C. Battistelli , T. Alonzi , A. Weisz , M. Tripodi , Cell Rep. 2016, 17, 799. 27732855 10.1016/j.celrep.2016.09.031

[38] J. Guduric‐Fuchs , A. O'connor , B. Camp , C. L. O'neill , R. J. Medina , D. A. Simpson , BMC Genomics 2012, 13, 357. 22849433 10.1186/1471-2164-13-357 PMC3532190

[39] M. J. Shurtleff , M. M. Temoche‐Diaz , K. V. Karfilis , S. Ri , R. Schekman , eLife 2016, 5, e19276. 27559612 10.7554/eLife.19276 PMC5047747

[40] L. Qu , J. Ding , C. Chen , Z.‐J. Wu , B. Liu , Y. Gao , W. Chen , F. Liu , W. Sun , X.‐F. Li , X. Wang , Y. Wang , Z.‐Y. Xu , L. Gao , Q. Yang , B. Xu , Y.‐M. Li , Z.‐Y. Fang , Z.‐P. Xu , Y. Bao , D.‐S. Wu , X. Miao , H.‐Y. Sun , Y.‐H. Sun , H.‐Y. Wang , L.‐H. Wang , Cancer Cell 2016, 29, 653. 27117758 10.1016/j.ccell.2016.03.004

[41] M. Khorasanizadeh , M. Eskian , E. W. Gelfand , N. Rezaei , Pharmacol. Ther. 2017, 174, 112. 28223227 10.1016/j.pharmthera.2017.02.024

[42] R. Newton , M. A. Giembycz , Br. J. Pharmacol. 2016, 173, 3405. 27646470 10.1111/bph.13628 PMC5120156

[43] G. Pelaia , G. Cuda , A. Vatrella , L. Gallelli , M. Caraglia , M. Marra , A. Abbruzzese , M. Caputi , R. Maselli , F. S. Costanzo , S. A. Marsico , J. Cell. Physiol. 2005, 202, 642. 15316926 10.1002/jcp.20169

[44] B. N. Lambrecht , H. Hammad , J. V. Fahy , Immunity 2019, 50, 975. 30995510 10.1016/j.immuni.2019.03.018

[45] T. M. Milillo , J. A. Gardella , Anal. Chem. 2008, 80, 4896. 18537271 10.1021/ac702640v

[46] Y. Ramos , B. St‐Onge , J.‐P. Blanchet , A. Smargiassi , J. Exposure Sci. Environ. Epidemiol. 2016, 26, 405. 10.1038/jes.2015.79 26648248

[47] Q. Xiong , Q. Ru , L. Chen , K. Yue , X. Tian , B. Ma , L. Liu , R. Wu , C. Xu , M. Pi , C. Li , J. Toxicol. Environ. Health, Part A 2015, 78, 443. 10.1080/15287394.2014.993490 25785558

[48] K. B. Cook , H. Kazan , K. Zuberi , Q. Morris , T. R. Hughes , Nucleic Acids Res. 2011, 39, D301. 21036867 10.1093/nar/gkq1069 PMC3013675

