# Supplementary material for: Fine Particulate Matter Induces Childhood Asthma Attacks via Extracellular Vesicle‐Packaged Let‐7i‐5p‐Mediated Modulation of the MAPK Signaling Pathway
Source: Adv Sci (Weinh). 2021 Nov 23;9(3):2102460. doi: 10.1002/advs.202102460 (PMC8787417; doi:10.1002/advs.202102460)

ADVANCED SCIENCE
Open Access

Supporting Information

for *Adv. Sci.*, DOI: 10.1002/advs.202102460

Fine particulate matter induces childhood asthma attacks via extracellular vesicle-packaged let-7i-5p-mediated modulation of the MAPK signaling pathway

Rui Zheng, Mulong Du*, Man Tian*, Zhaozhong Zhu, Chengcheng Wei, Haiyan Chu, Cong Gan, Jiayuan Liang, Renjie Xue, Fang Gao, Zhenguang Mao, Meilin Wang, Zhengdong Zhang*

Supplementary Materials and Methods

Supplementary Tables

Table S1. The cell viability of HBE cells after $PM_{2.5}$ treatment

Table S2. The characteristics of asthma cases and healthy controls

Table S3. The characteristics of children participated in RNA-Seq

Table S4. Sequences of primers used for RT-qPCR in the study

Table S5. Sequences of mimic, inhibitor and siRNA in the study

Supplementary Figures

Figure S1. The cytotoxic effect of normal HBE cells in coculture with $PM_{2.5}$-treated HBE cells.

Figure S2. The band intensity of EV-specific markers was assessed by Western blotting.

Figure S3. Fluorescent observation of HBE cells incubation with $PM_{2.5}$/NC-EVs pretreated with cytochalasin D**.**

Figure S4. The effect of inhibiting $PM_{2.5}$-EVs biogenesis on cytotoxicity in HBE cells.

Figure S5. The band intensity of contractile proteins was assessed by Western blotting.

Figure S6. Fluorescent observation of normal HBSMCs incubation with $PM_{2.5}$/NC-EVs pretreated with cytochalasin D**.**

Figure S7. Fluorescent observation of sensitive HBSMCs incubation with $PM_{2.5}$/NC-EVs pretreated with cytochalasin D**.**

Figure S8. Characterization of EVs derived from plasma.

Figure S9. The level of let-7i-5p in $PM_{2.5}$/NC-EVs.

Figure S10. Detection of the stability of EV-packaged let-7i-5p in plasma.

Figure S11. The expression correlation analysis of plasma EV-packaged let-7i-5p and individual's $PM_{2.5}$ exposure level.

Figure S12. The effect of let-7i-5p on recipient HBE cells and sensitive HBSMCs cellular phenotype.

Figure S13. $PM_{2.5}$-treated HBE cells secrete EV-packaged let-7i-5p into the recipient

cells.

Figure S14. The effect of blocking EV-packaged let-7i-5p on the recipient cellular phenotype.

Figure S15. The effect of EV-packaged let-7i-5p on the recipient cellular phenotype.

Figure S16. The ectopic expression of ELAVL1 in HBE cells.

Figure S17. The overlapped genes in three target prediction software programs.

Figure S18. The band intensity of MAPK pathway-related signaling molecules was assessed by Western blotting.

Figure S19. The protein levels of DUSP1 in recipient cells.

Figure S20. The protein levels of MAPK pathway-related signaling molecules after si-*DUSP1* transfection.

Figure S21. The effect of *DUSP1* on recipient HBE cells and sensitive HBSMCs cellular phenotype.

Figure S22. Expression of the contractile proteins α-SMA, SM-MHC and RhoA in lung tissues.

Figure S23. Expression of MAPK pathway-related signaling molecules in lung tissues.

Supplementary materials and methods

Cell lines and cell culture

The HBE cell lines and primary HBSMCs were purchased from Shanghai Institute of Cell Biology of Chinese Academy of Science (Shanghai, China). The cells were cultured in H-DMEM (Biological Industries, USA) supplemented with 10% heat-inactivated fetal bovine serum (FBS) (Biological Industries, USA), 100 μg/ml streptomycin (Gibco, USA) and 100 U/ml penicillin (Gibco, USA) in a humidified incubator containing of 5% CO_2 at 37 °C.

Establishment of the sensitive HBSMC model

Normal HBSMCs (passages 4-7) at 60-70% confluence were incubated with serum-free medium for 24 h before stimulation with 100 ng/ml recombinant human IL-13 (PeproTech, USA) for 24 h to generate sensitive HBSMCs. After treatment, the culture medium was collected for ELISA.

Cell Counting Kit (CCK)-8 assay

The indicated HBE cells were seeded in 96-well plates at a density of 3,000 cells/well and incubated for 24 h. Subsequently, cells were treated with stock $PM_{2.5}$ standard suspensions for 12 h, 24 h or 48 h or incubated with EVs for 24 h. A CCK-8 Kit (Dojindo Laboratories, Japan) and a microplate reader (Infinite M200 Pro, Switzerland) were used to measure the optical density at 450 nm (OD450).

Apoptosis assay and cell cycle analysis

For the apoptosis assay, the indicated HBE cells were processed with an Annexin V FITC Apoptosis Detection Kit (Dojindo Laboratories, Japan) and immediately analysed using a flow cytometer (Becton, Dickinson and Company, USA). For the

cell cycle assay, the indicated HBE cells were fixed with 3 ml of 70% ethanol at -20 °C for at least 18 h, stained with 500 µl of propidium iodide (PI) solution for 15 min, and analysed using flow cytometry.

Coculture assay

The indicated HBE cells were cocultured with HBE cells or sensitive HBSMCs at a ratio of 1:1 in a Transwell plate (0.4 µm pore size, Corning, USA) for 24 h, with recipient cells seeded in the lower chamber and the indicated HBE cells seeded in the upper chamber.

EV-packaged RNA extraction and RT-qPCR

Total RNA was extracted from cell lines with TRIzol reagent (Invitrogen, USA), and EV-packaged miRNAs were isolated from culture medium or plasma using an exoRNeasy Serum/Plasma Maxi Kit (Qiagen, Germany) according to the manufacturer's instructions. The cDNA templates were synthesized from miRNAs using Reverse Transcriptase XL (AMV) (TaKaRa, Japan), dNTP Mixture (TaKaRa, Japan) and Recombination RNase Inhibitor (TaKaRa, Japan), whereas cDNA templates were synthesized from mRNAs using a high-capacity cDNA reverse transcription kit (Invitrogen, USA). RT-qPCR for EV-packaged miRNAs or genes was conducted using a SYBR Green RT-PCR Kit (Vazyme Biotech, China) and a Roche Light Cycler 480 II system (Roche, Switzerland). In addition, U6, β-actin or *GAPDH* was used as the internal control. The relative expression levels of miRNAs and genes were calculated using the $2^{-\Delta Ct}$ method. The sequences of primers applied in this study are listed in Table S4.

Enzyme-linked immunosorbent assay (ELISA)

The level of eotaxin in the culture medium of HBSMCs was examined using a human Eotaxin 1 (Eotaxin 1/CCL11) ELISA kit (SenBeiJia Biotechnology, China). The IL-6 concentration in BALF from the animal model was measured with a mouse IL-6 ELISA kit (Hui Jia Biotechnology, China).

Determination of EV-packaged let-7i-5p stability in plasma

We subjected plasma samples from the cases and healthy controls to conditions including repeated freeze-thaw cycles (0, 2, 4, and 8 cycles) between -80 °C and room temperature or an incubation at room temperature for 0 h, 4 h, 8 h and 24 h to examine the stability of EV-packaged let-7i-5p in plasma.

Construction and transfection of the miRNA mimic/inhibitor and small interfering RNA (siRNA)

The let-7i-5p mimic and Cy3-labelled let-7i-5p mimic were synthesized and purified by RiboBio (China). The let-7i-5p inhibitor, si-ELAVL1 and si-*DUSP1* were constructed by GenePharma (China). The miRNA mimic/inhibitor or siRNA and the corresponding empty vector were transiently transfected into recipient cells using Lipofectamine 3000 reagent (Invitrogen, USA) according to the manufacturer's instructions. In addition, a lentiviral vector expressing let-7i-5p was constructed using the GV369 infection system (GeneChem, China). The sequences of the primers used for miRNA mimic/inhibitor or siRNA construction are shown in Table S5.

RIP assay

A Magna RIP kit (Millipore Magna, USA) was used to detect the interaction between let-7i-5p and ELAVL1. Briefly, lysates of $PM_{2.5}$/NC-treated HBE cells were incubated with RIP buffer containing anti-HuR/ELAVL1- or control IgG (ab172730, Abcam,

USA)-conjugated magnetic beads. Thereafter, the precipitated RNA fraction was analysed using RT-qPCR.

Colocalization of let-7i-5p and ELAVL1

The colocalization of let-7i-5p and ELAVL1 was evaluated using the immunofluorescence (IF) technique. HBE cells were transiently transfected with Cy3-labelled let-7i-5p mimic for 24 h and then fixed with 4% paraformaldehyde overnight. Subsequently, the indicated HBE cells were blocked with 1% BSA for 20 min and then incubated with anti-HuR/ELAVL1 for 2 h. The indicated HBE cells were incubated with the secondary antibody in the dark for 1 h and counterstained with DAPI. Images were acquired with a fluorescence microscope.

Luciferase reporter assay

We identified let-7i-5p target genes by amplifying the complete sequence of the *DUSP1* 3'-UTR and cloning it into the pmirGLO vector to construct the wild-type *DUSP1* plasmid; in addition, the seed sequence in the 3'-UTR of *DUSP1* was deleted, and the deletion construct was then inserted into the pmirGLO vector to construct the mutant *DUSP1* plasmid (Genebay Biotech Co., Ltd., China). The wild-type or mutant *DUSP1* luciferase plasmids and the let-7i-5p/NC mimic were cotransfected into recipient cells using Lipofectamine 3000 reagent. A Dual-Luciferase Reporter Assay System (Promega, USA) was used to measure luciferase activity.

Determination of airway smooth muscle contraction *in vitro*

Bronchi were harvested from the four groups of mice and were then stimulated with a continuous gradient concentration (1 nM–5.0 mM) of acetylcholine (Ach; Sigma, USA). The dose-response curve of Ach and tension was obtained and analysed using a

thermostatic perfusion system for *in vitro* tissues and organs (JADE Industry, China) with technical assistance provided by KeyGEN Biotech Co. Ltd.

Evaluation of EV-packaged let-7i-5p and *DUSP1* levels in BALF

The expression of EV-packaged let-7i-5p isolated from the BALF of a mouse model was evaluated using an exoRNeasy Serum/Plasma Maxi Kit (Qiagen, Germany). Levels of the *DUSP1* mRNA in BALF were determined after extraction using TRIzol reagent (Invitrogen, USA).

Histopathological assessment and immunohistochemistry (IHC)

Lung tissues were stained with haematoxylin and eosin (H&E). Based on alveolar hyperaemia, pulmonary haemorrhage, inflammatory cell infiltration and lung hyaline membrane formation and/or alveolar wall thickening, the lung injury score was assigned as follows: 1 point (< 25%), 2 points (25-50%), 3 points (50-75%) or 4 points (> 75%). After H&E staining, IHC was performed using anti-α-SMA (19245, CST, USA), anti-SM-MHC (ab53219, Abcam, USA), anti-RhoA (sc-418, Santa Cruz, USA), DUSP1 (ab217347, Abcam, USA), and the anti-phospho-MAPK family (9910, CST, USA) antibodies in lung tissues from the four groups.

URLs

RBPDB: http://rbpdb.ccbr.utoronto.ca/

DAVID: https://david-d.ncifcrf.gov/

Diana-MicroT: http://diana.imis.athena-innovation.gr/DianaTools/index.php

MiRwalk: http://mirwalk.umm.uni-heidelberg.de/

TargetScan: http://www.targetscan.org/

Supplementary Tables

Table S1. The cell viability of HBE cells after $PM_{2.5}$ treatment

**$PM_{2.5}$ (μg/ml)**	**Cell viability (%)**		
	12h	24h	48h
0	100.00	100.00	100.00
31.25	96.31 ± 1.10	93.18 ± 2.67*	96.04 ± 0.92*
62.5	94.73 ± 1.61*	93.44 ± 3.17*	97.02 ± 2.15
125	89.64 ± 2.79*	88.88 ± 1.22*	90.26 ± 0.95*
250	90.46 ± 0.77*	81.40 ± 1.14*	84.28 ± 0.30*
500	81.54 ± 2.71*	72.98 ± 0.93*	70.25 ± 1.14*
1,000	61.95 ± 0.63*	46.48 ± 1.14*	50.39 ± 1.28*
1,500	42.71 ± 1.64*	33.88 ± 1.91*	31.84 ± 1.13*

*Compared with the control group, $P < 0.05$.

Table S2. The characteristics of asthma cases and healthy controls

**Variables**	**Case (n = 55) %**	**Control (n = 55) %**	p^a
Age (years) (mean ± SD)	4.49 ± 1.9	4.19 ± 2.8	0.572
Sex			0.690
Male	37 (67.3)	34 (61.8)	
Female	18 (32.7)	21 (38.2)	
Log10-transformed plasma total IgE levels, IU/ml	2.34 ± 0.6	1.55 ± 0.5	< 0.001
Peripheral blood eosinophils[b], %	4.95 ± 3.8	1.42 ± 1.2	< 0.001
FEV1, predicted	1.30 ± 0.5	NA	
Log10-transformed FeNO, ppb	1.14 ± 0.3	NA	
$PM_{2.5}$ exposure, μg/m^3	55.69 ± 20.7	49.95 ± 24.7	0.189

IgE, immunoglobulin E; FEV1, forced expiratory volume in 1s; FeNO, fractional exhaled nitric oxide concentration; NA, not applicable.

[a] Student's *t*-test for age between cases and controls; Two-sided $\chi 2$ for sex between cases and controls.

[b] As percentage of total peripheral blood leukocytes.

Table S3. The characteristics of children participated in RNA-Seq

Cases (n=8)							Controls (n=8)				
**No**	**Age**	**Sex**	**Plasma total IgE levels[a]**	**Peripheral blood eosinophils[b]**	**FEV1, predicted**	**FeNO[c]**	**No**	**Age**	**Sex**	**Plasma total IgE levels[a]**	**Peripheral blood eosinophils[b]**
1	3	Male	3.26	12.80	0.68	0.90	1	1	Male	1.26	0.90
2	4	Male	2.73	10.80	0.94	1.36	2	3	Male	1.13	2.20
3	3	Male	3.02	5.70	0.83	1.08	3	8	Male	1.58	1.80
4	4	Male	2.43	5.30	0.95	1.46	4	5	Male	0.96	1.10
5	2	Male	2.83	7.20	0.58	0.78	5	3	Male	1.60	0.60
6	7	Female	3.08	8.00	1.60	1.41	6	3	Female	1.59	4.00
7	5	Female	3.04	7.50	1.30	1.32	7	2	Female	1.24	2.60
8	5	Male	2.30	9.60	1.22	1.66	8	4	Female	1.40	3.20

IgE, immunoglobulin E; FEV1, forced expiratory volume in 1s; FeNO, fractional exhaled nitric oxide concentration.

[a] Log10-transformed plasma total IgE levels, IU/ml.

[b] As percentage of total peripheral blood leukocytes.

[c] Log10-transformed FeNO, ppb.

Table S4. Sequences of primers used for RT-qPCR in the study

Gene name	Primer	Sequences (5'-3')
let-7i-5p	Forward	ACACTCCAGCTGGGTGAGGTAGTAGTTT
	Reverse	TGGTGTCGTGGAGTCG
	RT	CTCAACTGGTGTCGTGGAGTCGGCAATTCAGTTGAGAACAGCAC
U6	Forward	CGCTTCGGCAGCACATATACTAAAATTGGAAC
	Reverse	GCTTCACGAATTTGCGTGTCATCCTTGC
	RT	AAAATATGGAACGCTTCACG
ELAVL1	Forward	GGGTGACATCGGGAGAACG
	Reverse	CTGAACAGGCTTCGTAACTCAT
GAPDH	Forward	CCGGGAAACTGTGGCGTGATGG
	Reverse	AGGTGGAGGAGTGGGTGTCGCTGTT
DUSP1	Forward	AGTACCCCACTCTACGATCAGG
	Reverse	GAAGCGTGATACGCACTGC
β-actin	Forward	CATGTACGTTGCTATCCAGGC
	Reverse	CTCCTTAATGTCACGCACGAT
Mus-let-7i-5p	Forward	CGCGCGTGAGGTAGTAGTTTGT
	Reverse	AGTGCAGGGTCCGAGGTATT
	RT	GTCGTATCCAGTGCAGGGTCCGAGGTATTCGCACTGGATACGACAACAGC
Mus-U6	Forward	CGCTTCGGCAGCACATATAC
	Reverse	CACGAATTTGCGTGTCATCC

	RT	NNNNNN (random primer)
Mus-*DUSP1*	Forward	CGGATGCAGCTCCTGTAGTA
	Reverse	CCCAAGGCGTCAAGCATATC
Mus-*GAPDH*	Forward	ACTCTTCCACCTTCGATGCC
	Reverse	TGGGATAGGGCCTCTCTTGC

Table S5. Sequences of mimic, inhibitor and siRNA in the study

Gene name	Primer	Sequences (5'-3')
let-7i-5p mimic	Sense	UGAGGUAGUAGUUUGUGCUGUU
	Anti-sense	AACAGCACAAACUACUACCUCA
let-7i-5p inhibitor	Sense	AACAGCACAAACUACUACCUCA
si-ELAVL1	Sense	GAACGAAUUUGAUCGUCAATT
	Anti-sense	UUGACGAUCAAAUUCGUUCTT
si-*DUSP1*	Sense	CCAAUUGUCCCAACCAUUUTT
	Anti-sense	AAAUGGUUGGGACAAUUGGTT

Figure Legends

Figure S1. The cytotoxic effect of normal HBE cells in coculture with $PM_{2.5}$-treated HBE cells. $PM_{2.5}$/NC-treated HBE cells named as $PM_{2.5}$/NC-HBE cells. **(A)** Schematic illustration of normal HBE cells were cocultured with $PM_{2.5}$/NC-HBE cells in the Transwell plate (0.4 μm). **(B)** The cell viability of HBE was measured using a CCK-8 assay after co-cultivation with $PM_{2.5}$/NC-HBE cells. **(C)** The apoptosis of HBE cells was analyzed using flow cytometry after co-cultivation with $PM_{2.5}$/NC-HBE cells. **(D)** The cell cycle of HBE cells was tested using flow cytometry after co-cultivation with $PM_{2.5}$/NC-HBE cells. Statistical significance was assessed using two-tailed Student's *t*-test. Values represent means ± SD. $^{*}P < 0.05$.

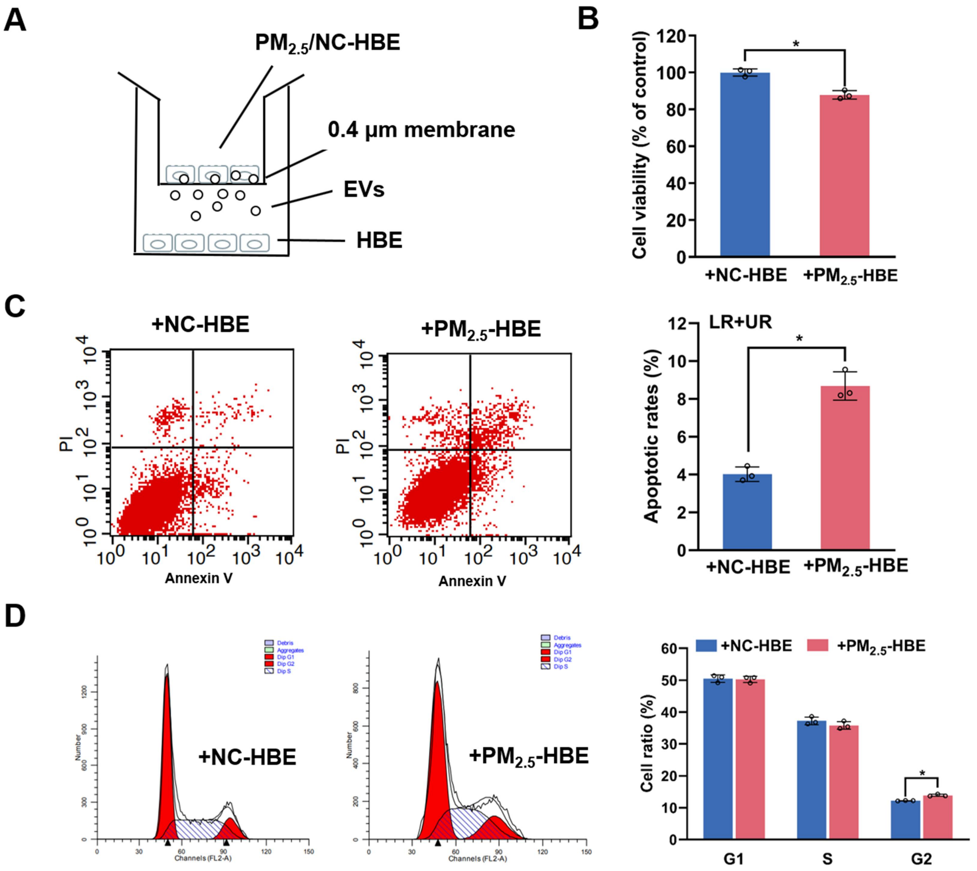

Figure S2. The band intensity of EV-specific markers was assessed by Western blotting. Statistical significance was assessed using two-tailed Student's *t*-test. Values represent means ± SD.

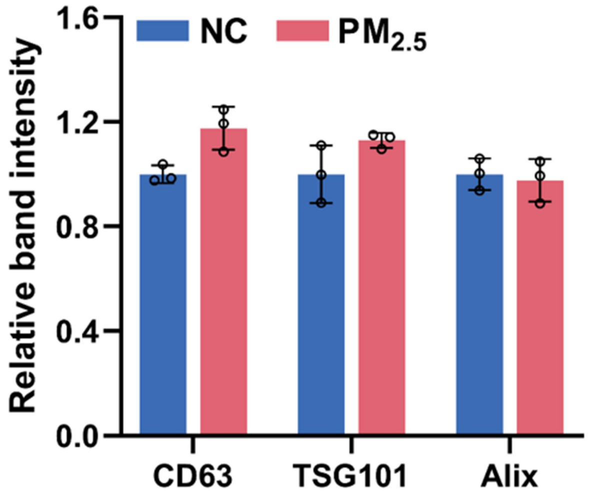

Figure S3. Fluorescent observation of HBE cells incubation with $PM_{2.5}$/NC-EVs pretreated with cytochalasin D. Representative images of HBE cells fluorescence after incubation with PKH67-labeled $PM_{2.5}$/NC-EVs pretreated with cytochalasin D (CytoD).

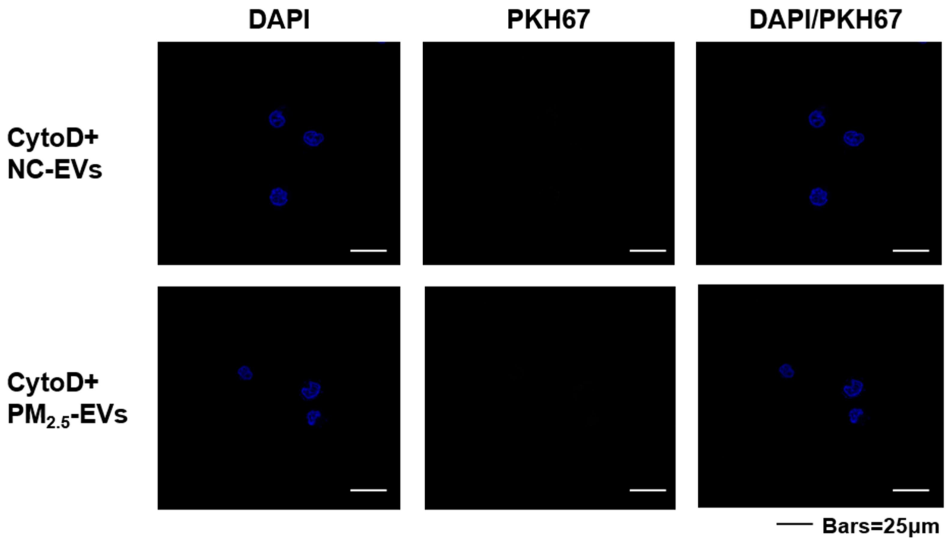

Figure S4. The effect of inhibiting $PM_{2.5}$-EVs biogenesis on cytotoxicity in HBE cells. HBE cells were treated with DMSO or EV secretion inhibitor GW4869, exposed to $PM_{2.5}$ and then isolated the corresponding EVs, namely $PM_{2.5}$-EVs-DMSO and $PM_{2.5}$-EVs-GW4869, respectively. Normal HBE cells were incubated with $PM_{2.5}$-EVs-DMSO/GW4869 to explore HBE cytotoxicity. **(A)** Graphical representation of HBE cells incubated with $PM_{2.5}$-EVs-DMSO/GW4869. **(B)** The viability of HBE cells was detected using a CCK-8 assay after incubation with $PM_{2.5}$-EVs-DMSO/GW4869. **(C)** The apoptosis of HBE cells was measured using flow cytometry after incubation with $PM_{2.5}$-EVs-DMSO/GW4869. **(D)** The cell cycle of HBE cells was tested using flow cytometry after incubation with $PM_{2.5}$-EVs-DMSO/GW4869. Statistical significance was assessed using two-tailed Student's *t*-test. Values represent means ± SD. $^{*}P < 0.05$.

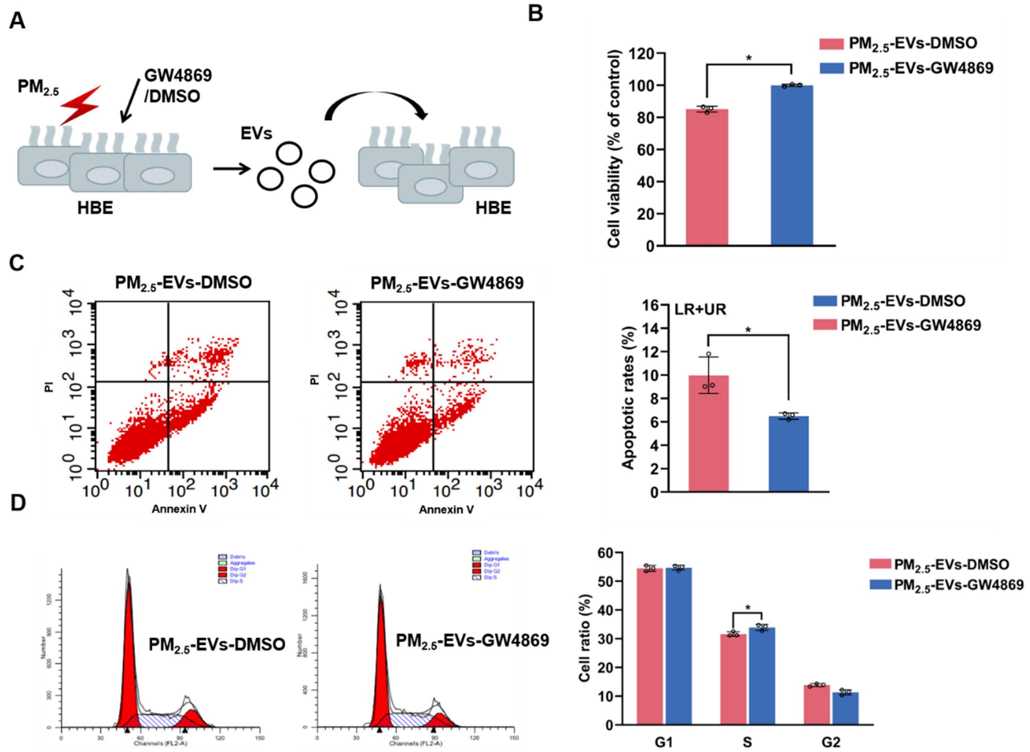

Figure S5. The band intensity of contractile proteins was assessed by Western blotting. (A) The band intensity of contractile proteins in normal HBSMCs cocultured with $PM_{2.5}$/NC-HBE cells. **(B)** The band intensity of contractile proteins in sensitive HBSMCs cocultured with $PM_{2.5}$/NC-HBE cells. **(C)** The band intensity of contractile proteins in sensitive HBSMCs incubated with $PM_{2.5}$/NC-EVs. **(D)** The band intensity of contractile proteins in sensitive HBSMCs incubated with $PM_{2.5}$/NC-EVs pretreated with DMSO/GW4869. Statistical significance was assessed using two-tailed Student's *t*-test. Values represent means ± SD. $^{*}P < 0.05$.

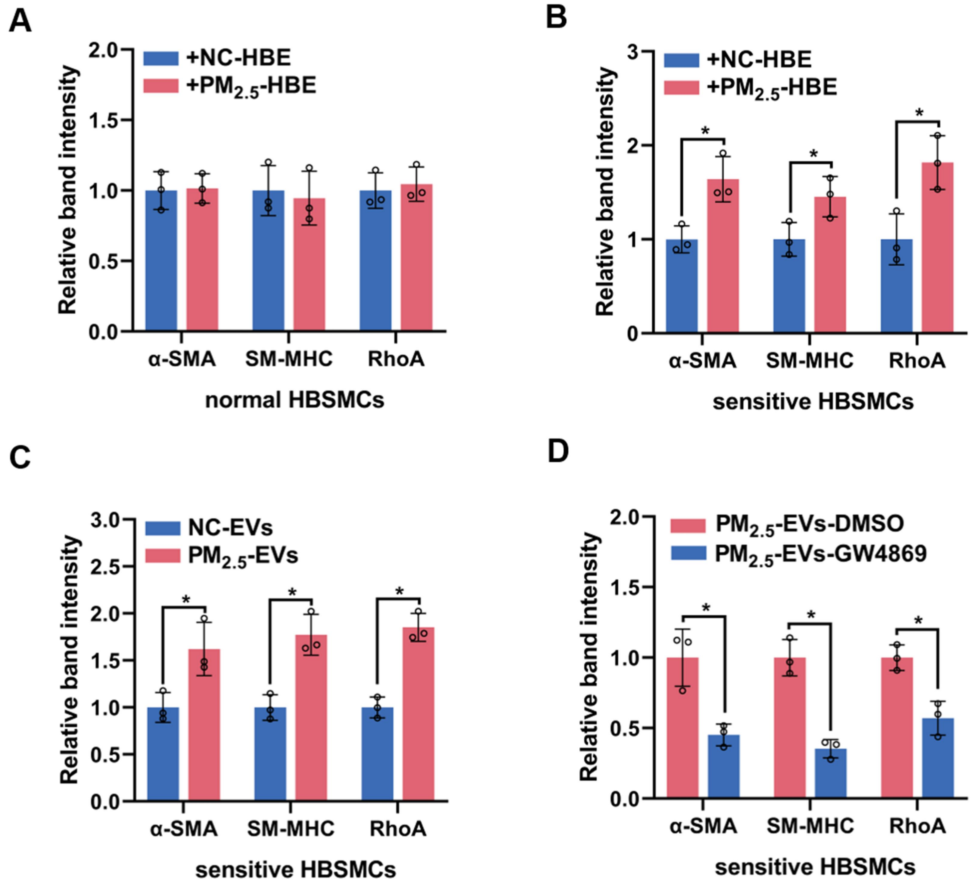

Figure S6. Fluorescent observation of normal HBSMCs incubation with $PM_{2.5}$/NC-EVs pretreated with cytochalasin D. Representative images of normal HBSMCs fluorescence after incubation with PKH67-labeled $PM_{2.5}$/NC-EVs pretreated with cytochalasin D (CytoD).

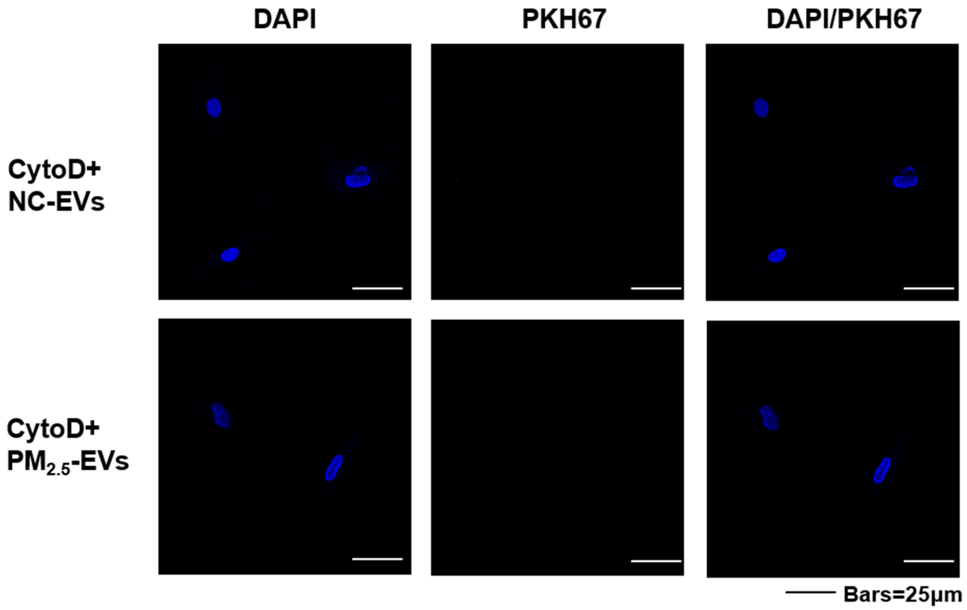

Figure S7. Fluorescent observation of sensitive HBSMCs incubation with $PM_{2.5}$/NC-EVs pretreated with cytochalasin D. Representative images of sensitive HBSMCs fluorescence after incubation with PKH67-labeled $PM_{2.5}$/NC-EVs pretreated with cytochalasin D (CytoD).

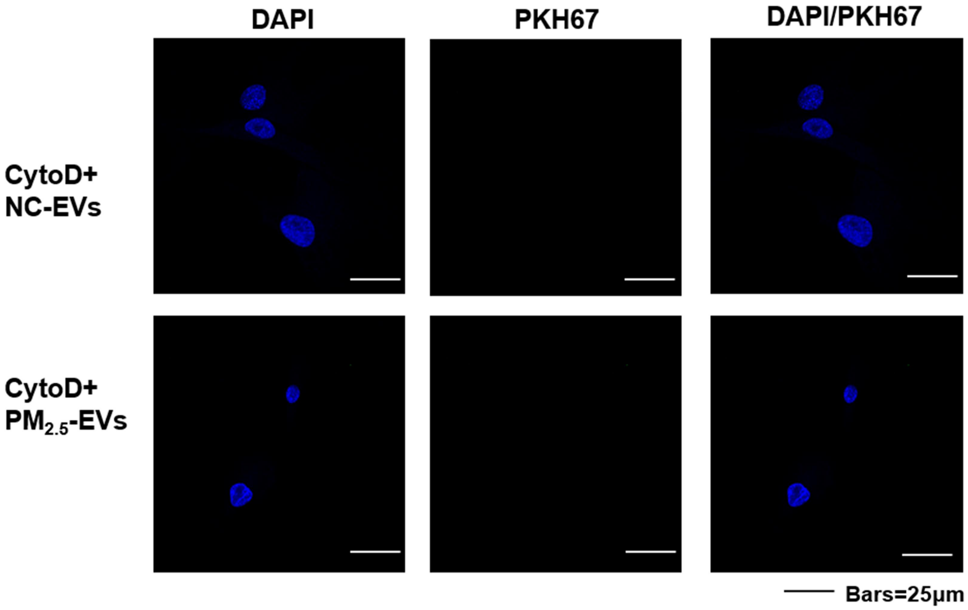

Figure S8. Characterization of EVs derived from plasma. (A) Purified EVs derived from plasma was confirmed by TEM. Scale bar, 100 nm. **(B)** The band intensity of EV-specific markers (CD63, TSG101 and Alix) was assessed by Western blotting. **(C)** The size distribution of EVs derived from plasma was analyzed by NanoFCM. Statistical significance was assessed using two-tailed Student's *t*-test. Values represent means ± SD. $^{*}P < 0.05$.

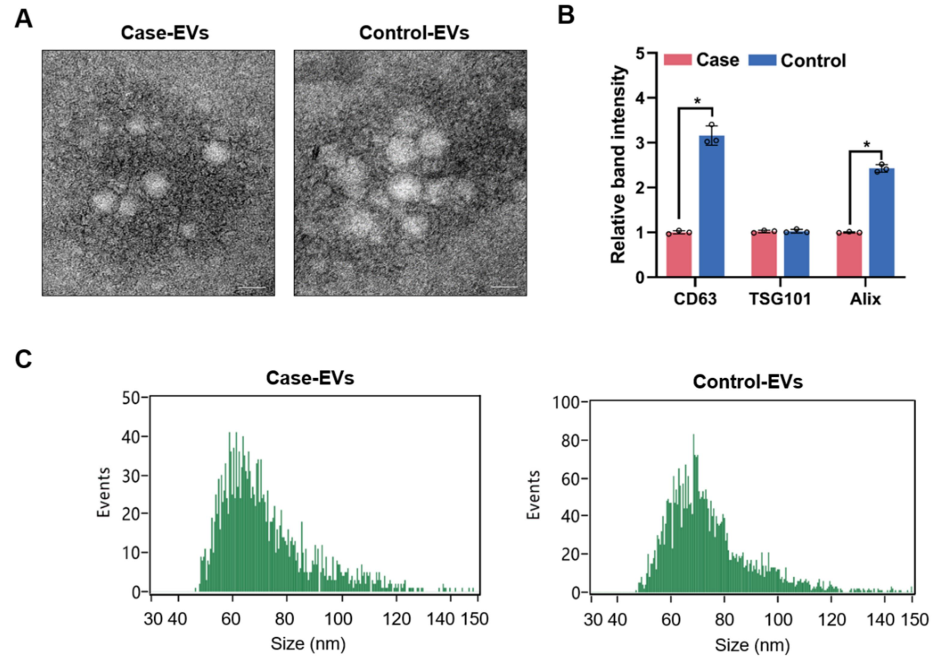

Figure S9. The level of let-7i-5p in $PM_{2.5}$/NC-EVs. Statistical significance was assessed using two-tailed Student's *t*-test. Values represent means ± SD. $^{*}P < 0.05$.

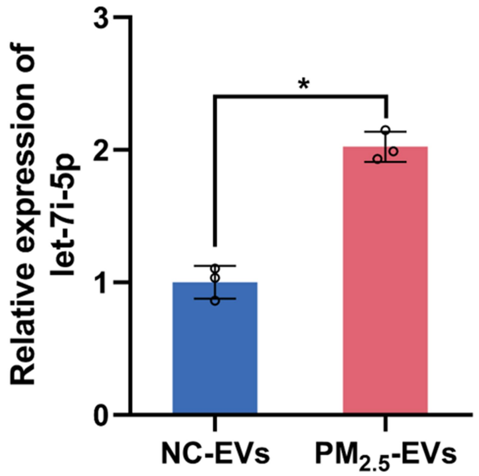

Figure S10. Detection of the stability of EV-packaged let-7i-5p in plasma. (A) The expression of EV-packaged let-7i-5p was measured after repeated thawing and freezing plasma repeatedly 0 cycle, 2 cycles, 4 cycles, and 8 cycles. **(B)** The expression of EV-packaged let-7i-5p was measured after placing plasma at room temperature for 0 h, 4 h, 8 h, and 24 h.

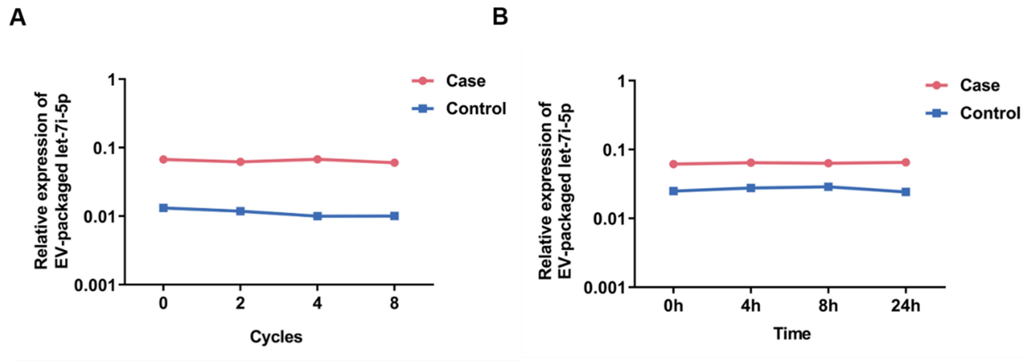

Figure S11. The expression correlation analysis of plasma EV-packaged let-7i-5p and individual's $PM_{2.5}$ exposure level. The correlation analysis was performed between plasma EV-packaged let-7i-5p and $PM_{2.5}$ exposure levels in healthy controls. Statistical significance was assessed using spearman correlation analysis.

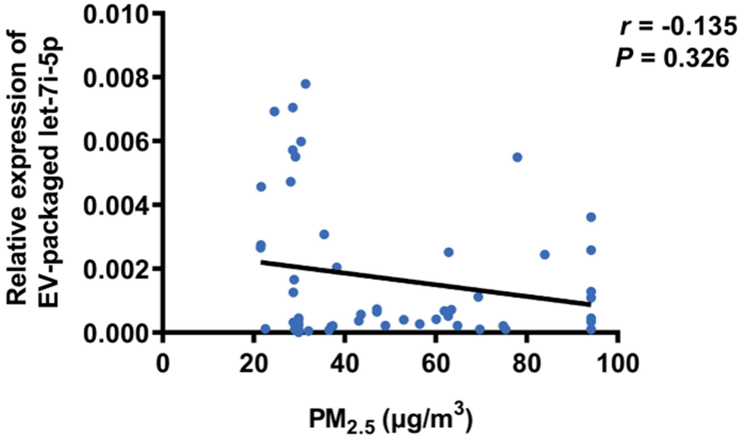

Figure S12. The effect of let-7i-5p on recipient HBE cells and sensitive HBSMCs cellular phenotype. HBE cells and sensitive HBSMCs were transfected with let-7i-5p mimic or NC mimic, namely let-7i-5p mimic and NC, respectively. **(A)** The ectopic expression of let-7i-5p in HBE cells was identified using RT-qPCR. **(B)** The cell viability of HBE cells was analyzed using a CCK-8 assay after transfection let-7i-5p mimic. **(C)** The apoptosis of HBE cells was tested using flow cytometry after transfection let-7i-5p mimic. **(D)** The cell cycle of HBE cells was detected using flow cytometry after transfection let-7i-5p mimic. **(E)** The ectopic expression of let-7i-5p in sensitive HBSMCs was identified using RT-qPCR. **(F)** Western blot analysis of the contractile proteins α-SMA, SM-MHC and RhoA in sensitive HBSMCs after transfection let-7i-5p mimic. The band intensity was assessed. Statistical significance was assessed using two-tailed Student's *t*-test. Values represent means ± SD. $^{*}P < 0.05$.

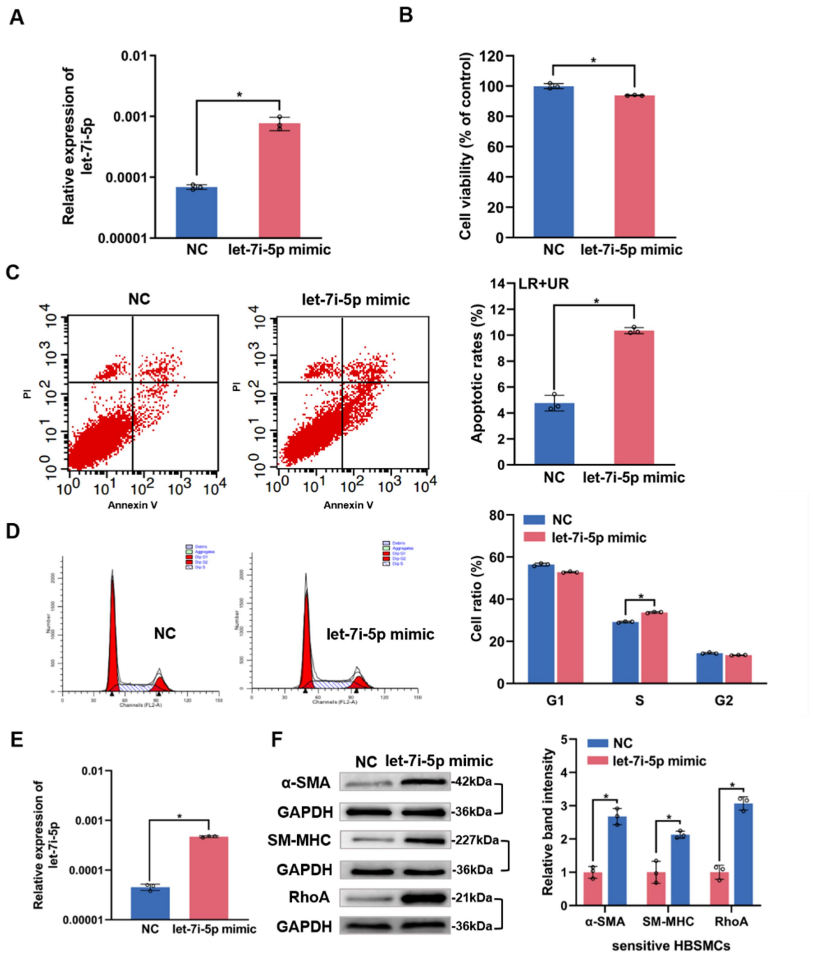

Figure S13. $PM_{2.5}$-treated HBE cells secrete EV-packaged let-7i-5p into the recipient cells. (A) HBE cells transiently transfected with a Cy3-tagged let-7i-5p (HBE-Cy3-let-7i-5p) mimic were cocultured with normal HBE cells or sensitive HBSMCs in the 0.4 µm Transwell plate. **(B)** Left panel: Schematic representation of the incubation of $PM_{2.5}$/NC-EVs with normal HBE cells. Right panel: The level of let-7i-5p in HBE cells after $PM_{2.5}$ treatment or incubation with $PM_{2.5}$/NC-EVs. **(C)** Left panel: Schematic representation of the incubation of $PM_{2.5}$/NC-EVs with sensitive HBSMCs. Right panel: The level of let-7i-5p in sensitive HBSMCs after $PM_{2.5}$ treatment or incubation with $PM_{2.5}$/NC-EVs. Statistical significance was assessed using two-tailed Student's *t*-test. Values represent means ± SD. $^{*}P < 0.05$.

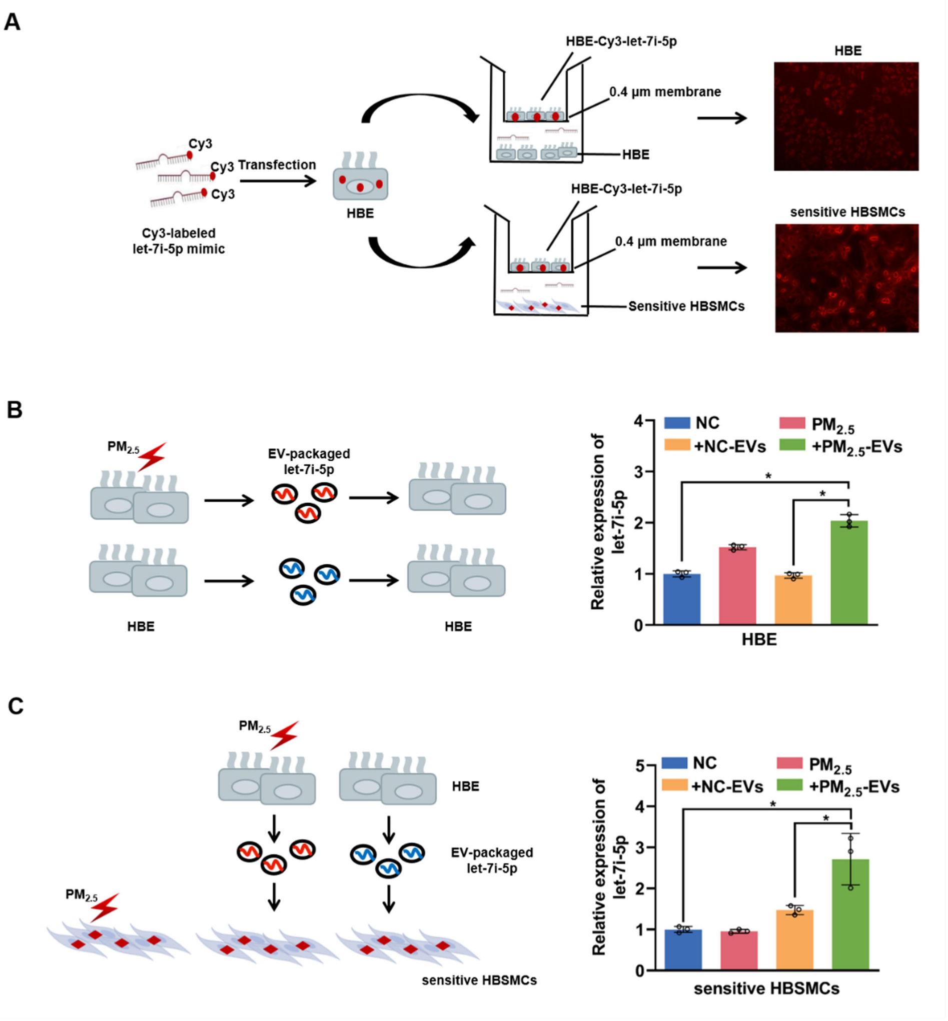

Figure S14. The effect of blocking EV-packaged let-7i-5p on the recipient cellular phenotype. HBE cells were transfected with NC- or let-7i-5p inhibitor, exposed to NC/$PM_{2.5}$ and then isolated the corresponding EVs, namely, NC-EVs, (NC+$PM_{2.5}$)-EVs and (Anti-let-7i-5p+$PM_{2.5}$)-EVs, respectively. Normal HBE cells were incubated with these EVs to explore the recipient cellular phenotype. **(A)** The diagram shows that normal HBE cells were incubated with NC-EVs, (NC+$PM_{2.5}$)-EVs or (Anti-let-7i-5p+$PM_{2.5}$)-EVs. **(B)** The diagram showed the sensitive HBSMCs were incubated with NC-EVs, (NC+$PM_{2.5}$)-EVs or (Anti-let-7i-5p+$PM_{2.5}$)-EVs. **(C)** The ectopic expression of let-7i-5p in HBE cells transfected with NC/let-7i-5p inhibitor was identified using RT-qPCR. **(D)** The ectopic expression of let-7i-5p in EVs was identified using RT-qPCR. **(E)** Representative images of the apoptosis of HBE cells incubated with NC-EVs, (NC+$PM_{2.5}$)-EVs or (Anti-let-7i-5p+$PM_{2.5}$)-EVs was assessed using flow cytometry. **(F)** Representative images of the cell cycle of HBE cells incubated with NC-EVs, (NC+$PM_{2.5}$)-EVs or (Anti-let-7i-5p+$PM_{2.5}$)-EVs was assessed using flow cytometry. Statistical significance was assessed using two-tailed Student's *t*-test. Values represent means ± SD. $^{*}P < 0.05$.

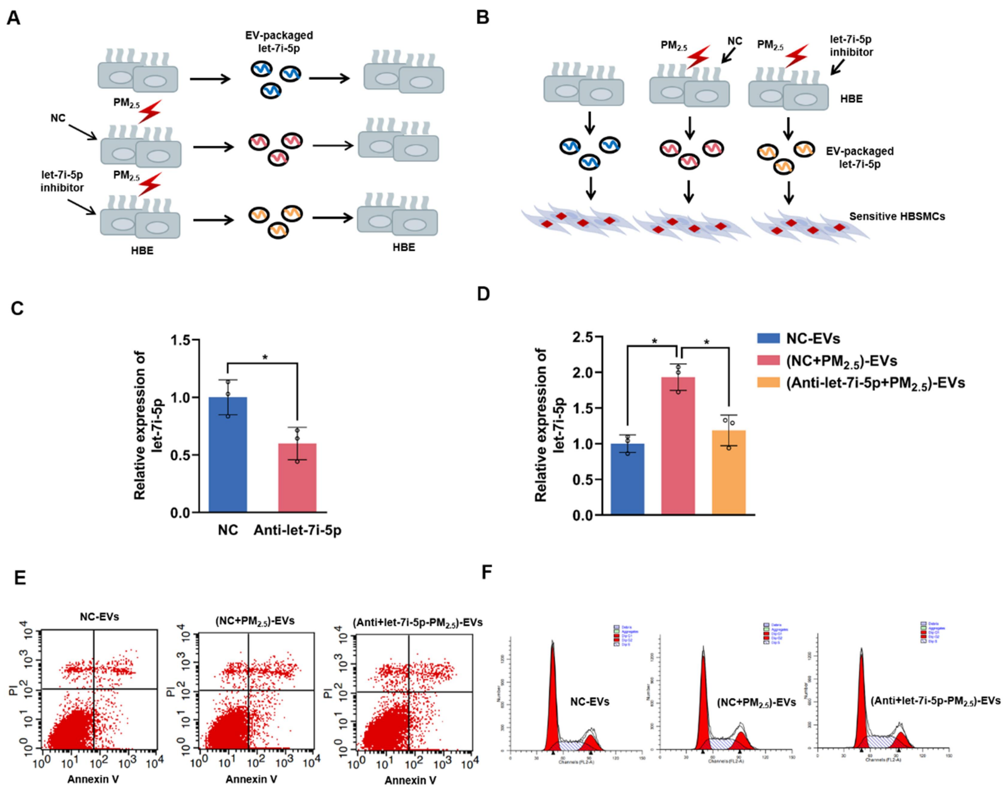

Figure S15. The effect of EV-packaged let-7i-5p on the recipient cellular phenotype. NC mimic- or let-7i-5p mimic-transfected normal HBE cells were incubated with $PM_{2.5}$/NC-EVs and designated NC-EVs, $PM_{2.5}$-EVs+NC and $PM_{2.5}$-EVs+let-7i-5p. **(A)** The diagram showed the NC mimic or let-7i-5p mimic-transfected normal HBE cells were incubated with $PM_{2.5}$/NC-EVs. **(B)** The viability of HBE cells was evaluated using a CCK-8 assay. **(C)** The apoptosis of HBE cells was assessed using flow cytometry. **(D)** The cell cycle of HBE cells was analyzed using flow cytometry. **(E)** The diagram showed the NC mimic or let-7i-5p mimic-transfected sensitive HBSMCs were incubated with $PM_{2.5}$/NC-EVs. **(F)** The levels of the contractile proteins α-SMA, SM-MHC and RhoA were measured using Western blotting. The band intensity was assessed. Statistical significance was assessed using two-tailed Student's *t*-test. Values represent means ± SD. $^{*}P < 0.05$.

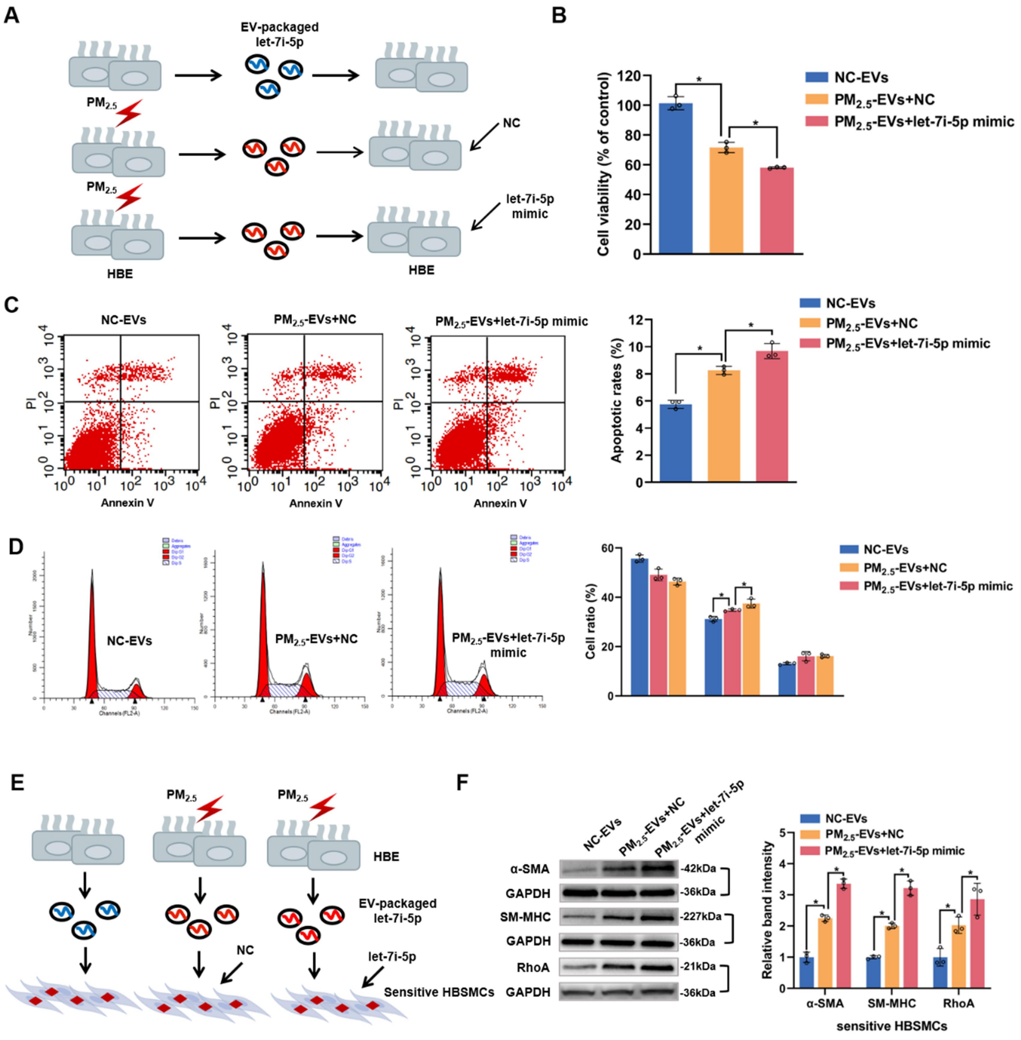

Figure S16. The ectopic expression of ELAVL1 in HBE cells. The expression levels of ELAVL1 in HBE cells after transfection with NC or si-ELAVL1 was measured using RT-qPCR **(A)** and Western blotting **(B)**. Statistical significance was assessed using two-tailed Student's *t*-test. Values represent means ± SD. $^{*}P < 0.05$.

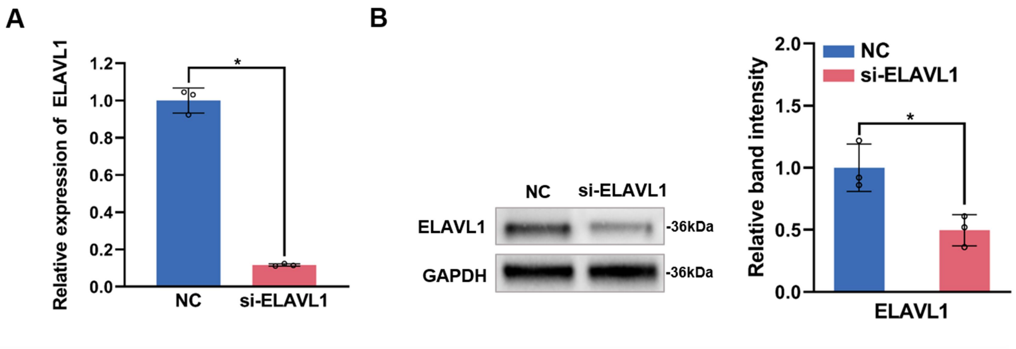

Figure S17. The overlapped genes in three target prediction software programs.

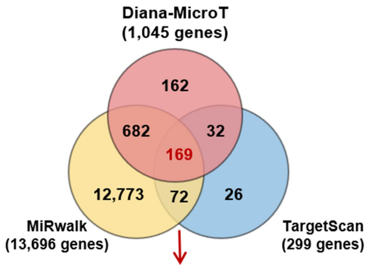

Overlapped genes									
ESPL1	TARBP2	CBX5	DYRK2	FRS2	NAP1L1	ADRB2	HAND1	DUSP1	CPEB4
RNF44	KATNAL1	EDN1	STARD13	PAG1	FNDC3A	SNX16	E2F5	MTDH	CDV3
ACVR2B	TSC22D2	LIMD1	CDC25A	NME6	TRAPPC1	PDE12	QARS	TAOK1	HIC2
ZNF280B	RBFOX2	CLP1	STX3	CCND1	PIGA	TXLNG	SMC1A	CCR7	GJC1
IGF2BP1	LIMD2	EPHA4	DPH3	TRIM71	TMPPE	MEX3A	GATM	TMOD2	MAPK6
PRTG	FAM103A1	IGDCC4	ARID3B	IGDCC3	AEN	IGF1R	TET3	SEMA4C	MGAT4A
RANBP2	SLC20A1	POLR2D	ONECUT2	ARID3A	TNFSF9	ZNF583	ZCCHC3	PLAGL2	FAS
CCNJ	LCOR	HIF1AN	SREK1IP1	FNIP1	ADIPOR2	CCND2	CHD4	GXYLT1	ABL2
RGS16	EDEM3	BTG2	PPP1R15B	MDM4	YOD1	LPGAT1	LBR	ACTA1	GALNT2
DZIP1	PRPF38B	COL4A1	C14orf28	SOCS4	GNG5	ZNF644	IRS2	LRIG2	NRAS
SEC14L1	PLEKHO1	POGZ	ARHGAP28	PEX11B	FAM104A	RNMT	WIPI2	IGF2BP3	HOXA9
PLEKHA8	AHCTF1	RRM2	SLC5A6	MAP4K3	MLLT10	ERCC6	DNA2	ZNF512B	BACH1
BEND4	CEP135	TMEM135	SMARCAD1	RDX	PXT1	CNTRL	PBX3	USP24	HOOK1
EGLN2	ACVR1C	EIF4G2	USP47	LGR4	DNAJA2	PDPR	GAN	FAM135A	SYNCRIP
RRAGD	LIN28B	PRDM1	LEPROTL1	BIN3	DUSP4	UTRN	FNDC3B	ABCC5	SENP2
PCGF3	NME4	ZNF200	CLDN12	BCAP29	WASL	PGRMC1	ZNF275	USP38	CASP3
RICTOR	C5orf51	MAP3K1	ZFYVE26	MED6	CALM1	DICER1	PTPRD	RNF38	

Figure S18. The band intensity of MAPK pathway-related signaling molecules was assessed by Western blotting. (A) The band intensity of MAPK pathway-related signaling molecules in NC mimic-, let-7i-5p mimic-, NC-EVs- or $PM_{2.5}$-EVs-treated HBE cells. **(B)** The band intensity of MAPK pathway-related signaling molecules in NC mimic-, let-7i-5p mimic-, NC-EVs- or $PM_{2.5}$-EVs-treated sensitive HBSMCs. Statistical significance was assessed using two-tailed Student's *t*-test. Values represent means ± SD. $^{*}P < 0.05$.

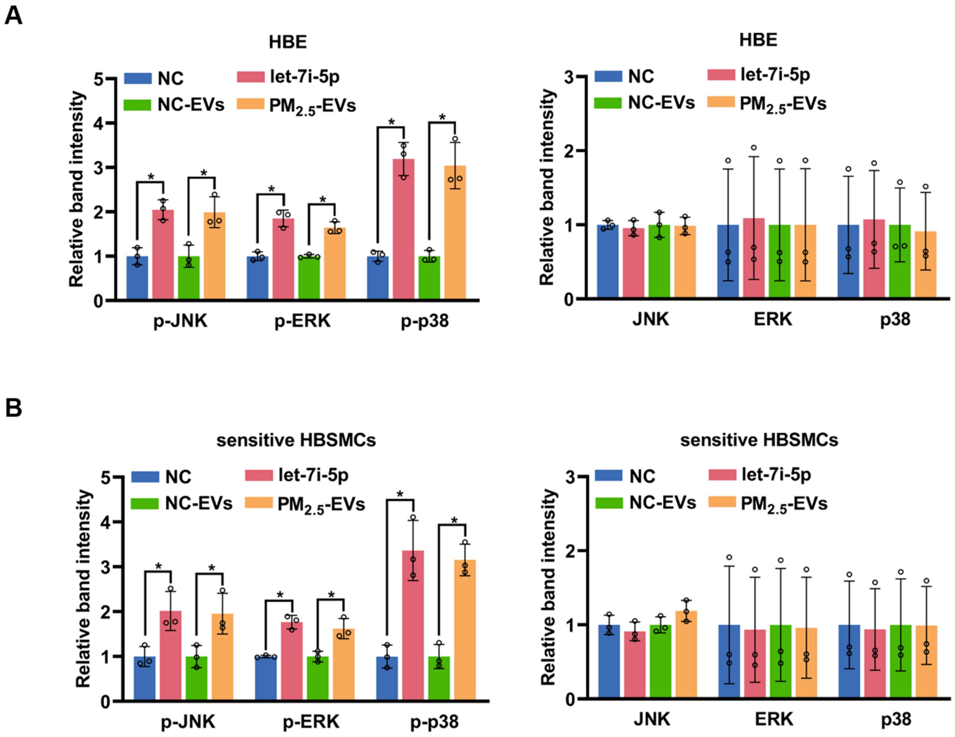

Figure S19. The protein levels of DUSP1 in recipient cells. **(A)** The protein expression of DUSP1 in NC mimic, let-7i-5p mimic, $PM_{2.5}$/NC-EVs-treated HBE cells was detected using Western blotting. **(B)** The protein expression of DUSP1 in NC mimic, let-7i-5p mimic, $PM_{2.5}$/NC-EV-treated sensitive HBSMCs was detected using Western blotting.

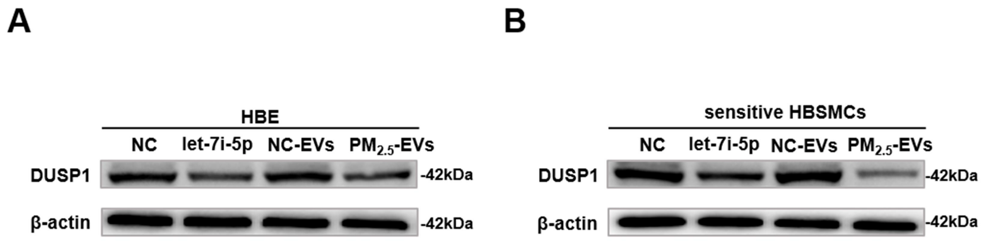

Figure S20. The protein levels of MAPK pathway-related signaling molecules after si-*DUSP1* transfection. The expression levels of *DUSP1* in HBE cells after transfection with NC or si-*DUPS1* was measured using RT-qPCR **(A)** and Western blotting **(B)**. The expression levels of *DUSP1* in sensitive HBSMCs after transfection with NC or si-*DUPS1* was measured using RT-qPCR **(C)** and Western blotting **(D)**. **(E)** The protein levels of MAPK pathway-related signaling molecules in NC vector or si-*DUSP1*-transfected recipient cells (HBE cells and sensitive HBSMCs) were detected using Western blotting. Statistical significance was assessed using two-tailed Student's *t*-test. Values represent means ± SD. $^{*}P < 0.05$.

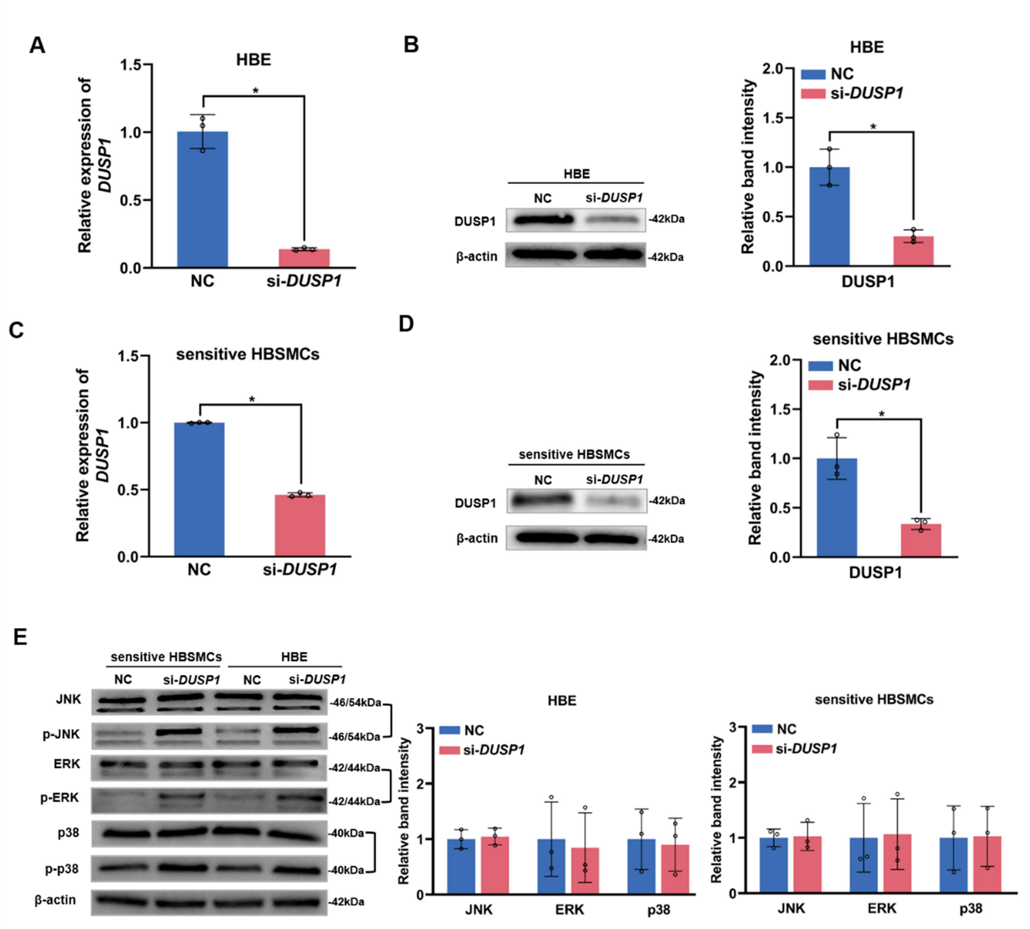

Figure S21. The effect of *DUSP1* on recipient HBE cells and sensitive HBSMCs cellular phenotype. HBE cells and sensitive HBSMCs were transfected with si-*DUSP1* or NC, namely si-*DUSP1* and NC, respectively. **(A)** The contractile proteins α-SMA, SM-MHC and RhoA in sensitive HBSMCs transfected with NC or si-*DUSP1* were tested using Western blotting. The band intensity was assessed. **(B)** The viability of HBE cells transfected with NC or si-*DUSP1* was evaluated using a CCK-8 assay. **(C)** The apoptosis of HBE cells transfected with NC or si-*DUSP1* was analyzed using flow cytometry. **(D)** The cell cycle of HBE cells transfected with NC or si-*DUSP1* was analyzed using flow cytometry. Statistical significance was assessed using two-tailed Student's t-test. Values represent means ± SD. $^{*}P < 0.05$.

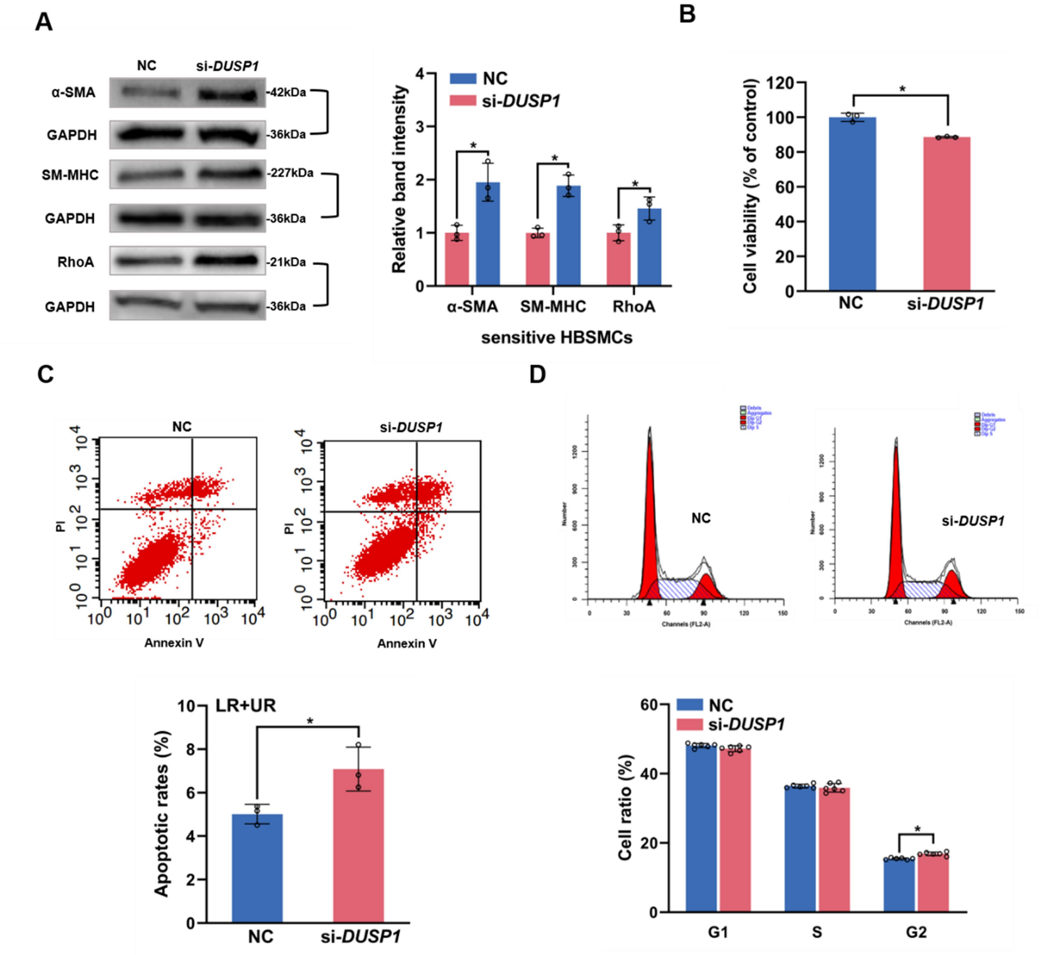

Figure S22. Expression of the contractile proteins α-SMA, SM-MHC and RhoA in lung tissues. Representative photographs and quantification of α-SMA **(A)**, SM-MHC **(B)**, and RhoA **(C)** immunostaining in lung tissues. Scale bar, 50 μm. Statistical significance was assessed using two-tailed Student's *t*-test. Values represent means ± SD. $^{*}P < 0.05$.

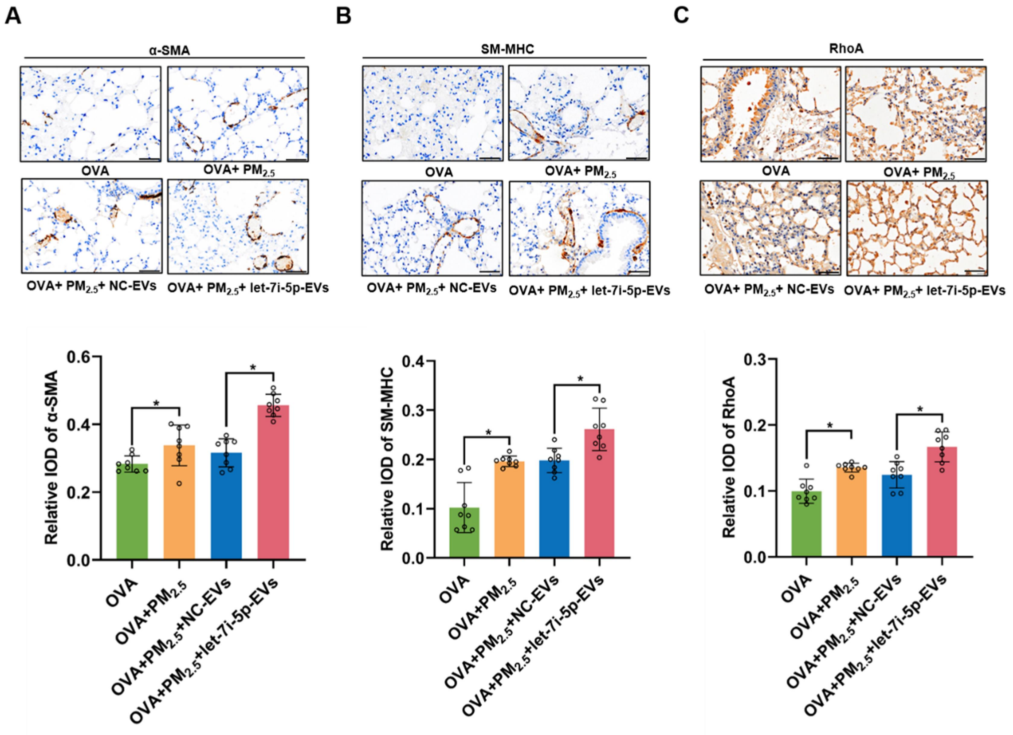

Figure S23. Expression of MAPK pathway-related signaling molecules in lung tissues. Representative photographs and quantification of p-JNK **(A)**, p-ERK **(B)**, and p-p38 **(C)** immunostaining in lung tissues. Scale bar, 50 μm. Statistical significance was assessed using two-tailed Student's *t*-test. Values represent means ± SD. $^{*}P <$ 0.05.

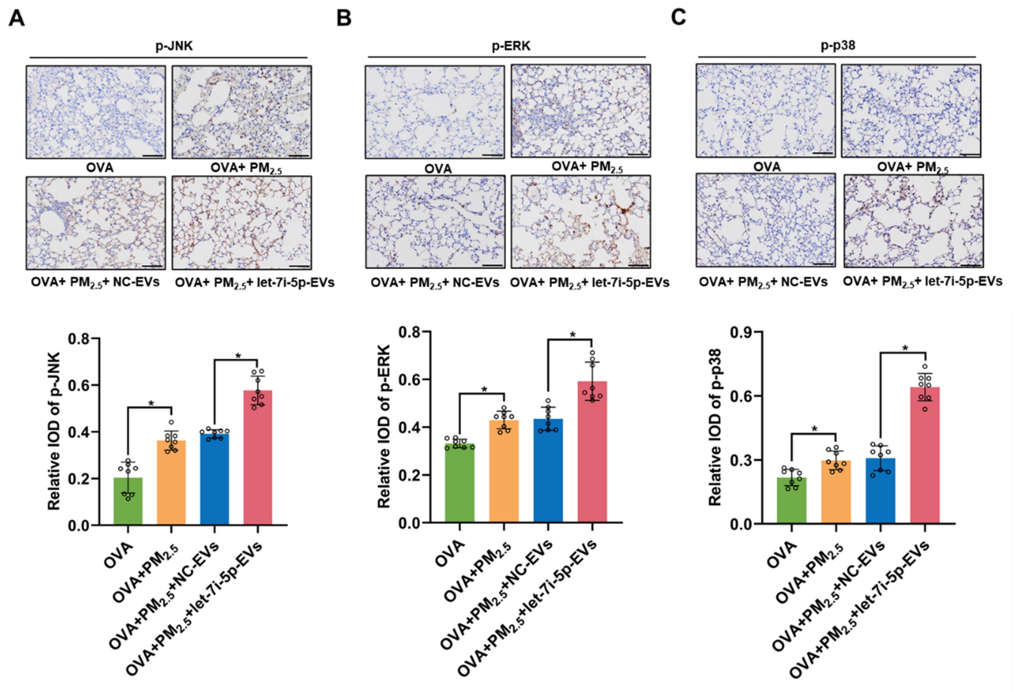

Supplement: Supplementary file 1 — Supporting Information [file ADVS-9-2102460-s001.pdf]
